# Inferring Characteristics of the Tumor Immune Microenvironment of Patients with HNSCC from Single-Cell Transcriptomics of Peripheral Blood

**DOI:** 10.1158/2767-9764.CRC-24-0092

**Published:** 2024-09-05

**Authors:** Yingying Cao, Tiangen Chang, Fiorella Schischlik, Kun Wang, Sanju Sinha, Sridhar Hannenhalli, Peng Jiang, Eytan Ruppin

**Affiliations:** 1 Cancer Data Science Laboratory, Center for Cancer Research, National Cancer Institute (NCI), National Institutes of Health (NIH), Bethesda, Maryland.; 2 Boehringer Ingelheim RCV Gmbh & Co KG, Vienna, Austria.; 3 NCI-Designated Cancer Center, Sanford Burnham Prebys Medical Discovery Institute, San Diego, California.

## Abstract

In this study, we explore the possibility of inferring characteristics of the tumor immune microenvironment from the blood. Specifically, we investigate two datasets of patients with head and neck squamous cell carcinoma with matched single-cell RNA sequencing (scRNA-seq) from peripheral blood mononuclear cells (PBMCs) and tumor tissues. Our analysis shows that the immune cell fractions and gene expression profiles of various immune cells within the tumor microenvironment can be inferred from the matched PBMC scRNA-seq data. We find that the established exhausted T-cell signature can be predicted from the blood and serve as a valuable prognostic blood biomarker of immunotherapy response. Additionally, our study reveals that the inferred ratio between tumor memory B- and regulatory T-cell fractions is predictive of immunotherapy response and is superior to the well-established cytolytic and exhausted T-cell signatures. These results highlight the promising potential of PBMC scRNA-seq in cancer immunotherapy and warrant, and will hopefully facilitate, further investigations on a larger scale. The code for predicting tumor immune microenvironment from PBMC scRNA-seq, *TIMEP*, is provided, offering other researchers the opportunity to investigate its prospective applications in various other indications.

**Significance:**

Our work offers a new and promising paradigm in liquid biopsies to unlock the power of blood single-cell transcriptomics in cancer immunotherapy.

## Introduction

The immune status in the tumor microenvironment (TME) plays a critical role in determining the progression of cancer and the efficiency of immunotherapy. Two important determinants of the immune status in the TME are the composition and gene expression of different immune cell types. In terms of cell abundance, it was found that FOXP3^+^ CD4^+^ regulatory T cells (Tregs) and CD8^+^ T cells are positively associated with patient prognosis in a cohort of patients with mixed human papillomavirus–positive/negative (HPV^+^/HPV^−^) head and neck squamous cell carcinoma (HNSCC; ref. 1). More recently, a positive association was reported between PD1^+^ TCF1^+^ CD8 in human HPV^+^ oropharyngeal squamous cell carcinoma and response to immune checkpoint blockade (ICB; ref. 2). Furthermore, a strong infiltration of CD8^+^ T cells is associated with favorable patient prognosis (3, 4), whereas a high infiltration of Treg correlates with poor prognosis in many solid tumors (5, 6). Similarly, a high CD8^+^/Tregs ratio is associated with favorable prognosis in multiple tumor types (7, 8). Additionally, tumor-infiltrating neutrophils, lymphocytes, and neutrophils-to-lymphocytes ratio have been found to be important markers of patient survival (9). Beyond cell abundance, the gene expression in the TME has been shown to be predictive of cancer ICB response, for example, by identifying gene expression signatures of T-cell dysfunction and exclusion (10) or by pairwise transcriptomic relations between immune checkpoint genes (ICGs; ref. 11).

The rapidly evolving single-cell sequencing technology now offers an unprecedented opportunity to analyze cell composition and gene expression of tumor cells as well as immune cells. For example, this technology can be used to study genomic and transcriptomic heterogeneity of tumors (12), construct a tumor immune cell atlas (13), chart cell–cell communication in the TME (14, 15), understand genomic and transcriptomic dynamics during cancer progression (16–18), and track tumor evolution (19). In the clinic, ideally, one would like to perform multiple tumor biopsies and employ single-cell transcriptomics to study the dynamics of the TME. However, for many tumors, such invasive procedures are prohibitive. In this study, we aim to determine the extent to which we can learn about the state of the TME from single-cell transcriptomics of immune cells in the blood.

Liquid biopsies have greatly advanced the field of clinical oncology and have become a focus of ongoing research for precision diagnosis and personalized cancer treatment. Current efforts have been focused on studying cell-free DNA (cfDNA), circulating tumor DNA (ctDNA), circulating tumor cells (CTCs), and methylation signatures [see (20) for a review]. Here, our focus is different and complementary, namely, to study single-cell transcriptomics of immune cells in the blood to learn about the immune status of the TME. Previous related studies have aimed to predict normal tissue gene expression from the *bulk* expression of white blood cells (WBCs). For example, analyzing matched whole blood/lung gene expression data from the Genotype-Tissue Expression (GTEx) project, a generalized linear regression model was shown to predict the expression levels of ∼18% of the genes in the lung from the blood (21). Using a Bayesian ridge regression–based method, the expression levels of 20% to 60% genes in 16 tissues could be predicted from the blood (22). Recent research has also shown that whole blood transcriptomes can predict tissue-specific expression levels for ∼60% of the genes on average across 32 tissues, and the inferred tissue-specific expression from the blood transcriptome can predict the disease state in some disorders (23).

Encouraged by our previous success with predicting tissue-specific expression from an individual’s blood bulk transcriptome (23), in this study we first aim to learn to what extent can one use single-cell blood transcriptome to learn about the cellular composition and gene expression of various immune cell types within an individual’s TME. Second, based on that, we aim to learn if we can use the inferred TME immune state to predict ICB response, and furthermore, if that leads to more accurate predictions than predictors that are built directly on the blood information without inferring the TME immune state.

To this end, we first analyzed a dataset of tumor blood–matched single-cell RNA sequencing (scRNA-seq) data for HNSCC from 26 patients. We hypothesized that immune cells in the peripheral blood mononuclear cells (PBMCs) bear information on the immune state in the TME, due to ongoing circulation of both immune cells and immune signaling molecules like cytokines between the blood and the TME. Indeed, many previous studies have shown that the number and function of both innate and adaptive immune cells in the blood are altered in patients with cancer (24–26). Additionally, it has been found that the dynamics of circulating immune cell phenotypes reflect interactions between tumor and immune cells during immunotherapy (27). Furthermore, numerous studies have also reported that changes in blood immune cell composition, particularly in neutrophils-to-lymphocytes ratio, are associated with poor survival outcomes and response to immunotherapy in many types of solid tumors (28). Indeed, we find that the immune cell fractions (ICFs) of major immune cell types in the TME can be predicted from PBMC scRNA-seq as they are correlated with ICFs of certain cell types in the latter. ICF is a proxy for cell abundance and represents the fraction of cells of a given immune cell type of the total number of all viable CD45^+^ immune cells in the same sample which includes almost all immunologic and hematologic cells except for mature erythrocytes and platelets (29, 30). Additionally, we find that in different immune cell types in the TME the expression levels of 17% to 47% expressed genes can be predicted from the patient’s blood.

We then analyzed a second dataset of patients with HNSCC including both patient pretreatment blood scRNA-seq data and ICB response information. We set to study if we can predict ICB response based on patients’ PBMC scRNA-seq data. We find that the well-established exhausted T-cell signature in the TME can be inferred from the blood and the inferred exhausted T-cell score can further predict immunotherapy response in HNSCC. In addition, we identify a new immune signature named ICFR^∗^, which reflects the blood-inferred TME ICF ratio of (B_memory_ − Treg)/(B_memory_ + Treg). The inferred ICFR^∗^ scores in the TME are the most predictive of patients’ response to ICB treatment both in terms of tumor RECIST criteria and pathologic response in this dataset and importantly, are further validated in an independent large-scale bulk expression HNSCC dataset. Here, RECIST response is a measure of best overall response on serial radiologic analysis (31), whereas pathologic response is defined as “whether patients have 90% or less viable tumor after the ICB treatment” (32).

## Materials and Methods

### scRNA-seq data analysis

Seurat (v4.0.1, R package; RRID: SCR_016341; ref. 33) was used for data processing and cell clustering. Cells with insufficient number of genes (< 200) or greater than 20% of unique molecular identifiers (UMIs) mapped to mitochondrial genes were removed. Data integration across different samples was accomplished with Harmony (34). The “*NormalizeData*” function with parameters: *normalization.method =* “*LogNormalize*” and *scale.factor =* 10,000 was applied to normalize the expression levels of genes in each single cell. The “*FindVariableFeatures*” function with the “*vst*” method was utilized to identify 2,000 highly variable genes. The “*ScaleData*” function with default parameters was used to scale and center gene expression matrices. To perform clustering, principal component analysis (PCA) dimensionality reduction was first conducted with “*RunPCA*” function and the first 20 principal components were selected to construct the shared nearest neighbor (SNN) graph with “*FindNeighbors*” function and then clusters were determined using the Louvain algorithm with “*FindClusters*” function. CellTypist (35) with the default model (*Immune_All_Low.pkl*) and majority voting refinement was used to do automatic cell type annotation with the known cell type labels (in total 19 cell types) to predict the identities of cell clusters. Then, we manually checked whether the annotations are reliable by examining the top ranked differentially expressed genes of each cluster which were obtained with “*FindAllMarkers*” function with default parameters but with set *min.pct = 0.25*. The uniform manifold approximation and projection (UMAP) was finally applied to visualize the single-cell transcriptional profile in the 2D space.

### Differential cell abundance and gene expression analysis

Differential cell abundance analysis was conducted with edgeR (RRID: SCR_012802; ref. 36) to identify which cell types were differentially enriched in tumor tissue or in the blood. “*estimateDisp()*” function was used to estimate the negative binomial dispersion for each cluster. “*glmQLFit()*” function was used to estimate the quasi-likelihood dispersion. “*glmQLFTest()*” function was used to test for differences in abundance between tumor tissue and blood with FDR < 0.05.

In this study, the gene expression of a specific immune cell type was defined as “pseudo-bulk” expression profiles generated by summing counts together for all individual cells of the same cell type in a sample. “Pseudo-bulk” samples with insufficient cells (< 10) were removed. As a result, 10 cell types [cycling T cells, cytotoxic T cells, dendritic cells (DCs), helper T cells, memory B cells, monocytes, naïve B cells, NK cells, plasma cells, and Tregs] were retained for differential gene expression analysis. “*pseudoBulkDGE()*” function from scran (v1.22.1, R package; RRID: SCR_016944) was used to perform the differential gene expression analysis to find significantly up and downregulated genes with FDR < 0.05 and log_2_ fold change (FC) = 0. This approach generated for each cell type a list of genes over- and underexpressed in the tumor compared with the peripheral blood. Gene Ontology (GO) enrichment analyses of the resulting gene lists were performed with R package *clusterProfiler* (v 4.2.2; RRID: SCR_016884).

### Machine learning for the prediction of the ICFs in the TME

To predict the ICFs in the TME from the blood, for each immune cell type in the TME, we first identified the most predictive cell type in the blood using the highest Pearson correlation coefficient (*r*_max_) of ICFs with the target cell types in the TME. If the correlation between a cell type in the TME with the identical cell type in the blood was higher than *r*_max_ × 0.9, then the identical cell type was used as the most predictive cell type. To reduce the impact of potential sampling bias, the correlation was calculated using 1,000-replicate bootstrapping.

Then, for each cell type in the TME, we constructed a one-variable linear regression baseline model by using the ICF of the corresponding most predictive cell type in the blood (as the only variable; model 1). Additionally, we constructed five two-variable linear regression models by using the ICF of the corresponding most predictive cell type in the blood (as the first variable) and one of the five clinical variables (as the second variable; models 2–6).

$$\text{Model}\;1:\;\hat{\text{ICF}}\left(i,\text{TME}\right)={⍺}_{1}*\text{ICF}\left(c_{i},\text{PBMCs}\right)\;+\;⍷$$

$$\text{Model}\;2:\hat{\;\text{ICF}}\left(i,\text{TME}\right)={⍺}_{1}*\text{ICF}\left(c_{i},\text{PBMCs}\right)+{⍺}_{2}*\text{Alcohol Use}\;+\;⍷$$

$$\text{Model}\;3:\;\hat{\text{ICF}}\left(i,\text{TME}\right)={⍺}_{1}*\text{ICF}\left(c_{i},\text{PBMCs}\right)+{⍺}_{2}*\text{Tobacco Use}\;+\;⍷$$

$$\text{Model}\;4:\;\hat{\text{ICF}}\left(i,\text{TME}\right)={⍺}_{1}*\text{ICF}\left(c_{i},\text{PBMCs}\right)+{⍺}_{2}*\text{HPV infection}\;+\;⍷$$

$$\text{Model}\;5:\;\hat{\text{ICF}}\left(i,\text{TME}\right)={⍺}_{1}*\text{ICF}\left(c_{i},\text{PBMCs}\right)+{⍺}_{2}*\text{Age}\;+\;⍷$$

$$\text{Model}\;6:\;\hat{\text{ICF}}\left(i,\text{TME}\right)={⍺}_{1}*\text{ICF}\left(c_{i},\text{PBMCs}\right)+{⍺}_{2}*\text{Sex}\;+\;⍷$$

in which *i* is the *i*th immune cell type in the TME; *c*_*i*_ is the corresponding most predictive cell type in the blood.

To reduce the risk of overfitting, elastic-net regularization was used during model training. Finally, for each cell type, among all six models we selected the model with the best performance as the final model. To perform cross-validation, the data were randomly split (80%: 20%) to do the training and testing for 1,000 replicates and the results were computed and analyzed over all 1,000 replicates.

The ICF of a specific immune cell type in the TME is defined predictable when the mean Pearson correlation coefficients between the predicted ICFs and the true ICFs on the 20% test sets are greater than 0.3 (*r* > 0.3), mean normalized mean absolute error less than 1 (NMAE < 1) with a Benjamini–Hochberg adjusted *P* value < 0.05 (FDR < 0.05) over 1,000 replicates. More details are in the following section of “*Statistical test to identify predictable ICFs and genes*.”

For model evaluation on the validation dataset and model application in predicting patient ICB response, the mean values of the parameters (i.e., coefficients and intercepts of the linear models) of the final models trained over 1,000 replicates were used.

### Machine learning for the prediction of the gene expression levels in immune cells in the TME

Similar to the prediction of ICFs, we used one- and two-variable linear regression models to predict expression levels of expressed genes in different immune cell types in the TME. In this case, “expressed genes” are defined as genes that have non-zero expression values in more than half of the samples. Specifically, for each immune cell type in the TME, we first identified the most predictive cell type in the blood based on the Pearson correlation coefficient of the overall gene expression levels across samples. Then, we used six linear regression models to predict the expression level of a specific gene in a specific immune cell type in the TME.

$$\text{Model}\;1:\;\hat{\mathrm{Exp}}\left(i,g_{j},\text{TME}\right)={⍺}_{1}*\mathrm{Exp}\left(c_{i},g_{j},\text{PBMCs}\right)\;+\;⍷$$

$$\text{Model}\;2:\hat{\mathrm{Exp}}\left(i,g_{j},\text{TME}\right)={⍺}_{1}*\mathrm{Exp}\left(c_{i},g_{j},\text{PBMCs}\right)+{⍺}_{2}*\text{Alcohol Use}\;+⍷$$

$$\text{Model}\;3:\;\hat{\mathrm{Exp}}\left(i,g_{j},\text{TME}\right)={⍺}_{1}*\mathrm{Exp}\left(c_{i},g_{j},\text{PBMCs}\right)+{⍺}_{2}*\text{Tobacco Use}\;+\;⍷$$

$$\text{Model}\;4:\;\hat{\mathrm{Exp}}\left(i,g_{j},\text{TME}\right)={⍺}_{1}*\mathrm{Exp}\left(c_{i},g_{j},\text{PBMCs}\right)+{⍺}_{2}*\text{HPV infection}\;+\;⍷$$

$$\text{Model}\;5:\;\hat{\mathrm{Exp}}\left(i,g_{j},\text{TME}\right)={⍺}_{1}*\mathrm{Exp}\left(c_{i},g_{j},\text{PBMCs}\right)+{⍺}_{2}*\text{Age}\;+\;⍷$$

$$\text{Model}\;6:\;\hat{\mathrm{Exp}}\left(i,g_{j},\text{TME}\right)={⍺}_{1}*\mathrm{Exp}\left(c_{i},g_{j},\text{PBMCs}\right)+{⍺}_{2}*\text{Sex}\;+\;⍷$$

in which *i* is the *i*th immune cell type in tumor tissue; g_j_ is the *j *th expressed gene; and c_i_ is the corresponding most predictive cell type in PBMCs identified by correlation of overall gene expression levels with i.

Elastic-net regularization was used during model training. Finally, for each gene, among all the six models we selected the model with the best performance as the final model. The results were computed, evaluated, and analyzed over 1,000 replicates.

### Statistical test to identify predictable ICFs and genes

Pearson correlation coefficients between the predicted and true values of the ICF/gene expression in the TME on the test data over 1,000 replicates were calculated for each cell type/gene and were tested against the null hypothesis of:

H01: The mean value of these Pearson correlation coefficients is ≤ 0.3.

Similarly, the normalized mean absolute errors between the predicted and true values over all 1,000 replicates were tested against the null hypothesis of:

H02: The mean value of these normalized mean absolute errors is ≥ 1.

We applied FDR < 0.05 to both hypotheses (H01 and H02) to select the predictable ICFs and genes.

With regard to identifying the clinical variables that have the highest and significant contribution to increasing the predictive power of each model, we compared results from models 2 to 6 with the result from model 1 using the Mood median test.

### Functional enrichment analysis

R package clusterProfiler (v 4.2.2; RRID: SCR_016884) was used to perform functional enrichment analysis of predictable gene lists including enrichment analysis of GO (RRID: SCR_002811) and enrichment analysis of Kyoto Encyclopedia of Genes and Genomes (RRID: SCR_012773), as well as enrichment analysis of hallmarks gene sets and Reactome subset of canonical pathways downloaded from MSigDB (http://www.gsea-msigdb.org/; RRID: SCR_016863).

### Identification of blood-predictive signatures for the prediction of ICB response

To identify the potential immune signatures that can be learned from the blood and are predictive of the ICB response of patients with HNSCC, we comprehensively examined three categories of candidate immune information in the blood and in the TME, respectively, i.e., ICGs, ICFs, and the ratios between immune cell fractions (ICFRs; see Supplementary Material SM2 for comprehensive lists of them). All the three categories of candidate immune information in the TME were predicted from the blood scRNA-seq data using the machine learning models described above. In terms of ICB response, we conducted a thorough evaluation using multiple metrics wherever relevant data were available. These metrics included the RECIST response, which measures the best overall response on serial radiologic analysis (31); the pathologic response, defined as “whether patients have 90% or less viable tumor after the ICB treatment” (32); overall survival (OS); and progression-free survival (PFS). Note that among the total 30 patients in the Luoma and colleagues cohort, 17 and 29 of them have the RECIST response and pathologic response data available, respectively. In the GSE159067 cohort, the pathologic response was not available.

With regard to ICGs, in total, 79 ICGs were collected from literature review (11, 37–40). We first checked the expression levels of them in different immune cell types in the blood. There are 219 expressed pairs in the blood, among which 31 pairs in the TME can be predicted from the blood (with criteria of *r* > 0.3 and FDR < 0.05 on the training dataset; *r* > 0.3 on the validation dataset). With regard to ICFs, nine are present in the blood (ICF > 0) and six in the TME can be predicted from the blood (with criteria of *r* > 0.3 and FDR < 0.05 on the training dataset; *r* > 0.3 on the validation dataset; Fig. 2C). With regard to ICFRs, there are in total 3,476 different combinations in the form of (ICF_C1 ± ICF_C2)/(ICF_C3 + ICF_C4) in the blood, in which C1, C2, C3, and C4 are immune cell types, and 55 in the TME are significantly predictable (with criteria of *r* > 0.3; *P* < 0.05; ρ > 0.3; *P* < 0.05 in both the training dataset and the validation dataset). As C1, C2, C3, and C4 are not necessarily different from each other, the form of (ICF_C1 ± ICF_C2)/(ICF_C3 + ICF_C4) can actually include all the following forms: ICF_C1/ICF_C2, ICF_C1/(ICF_C2 + ICF_C3), (ICF_C1 ± ICF_C2)/ICF_C3, and (ICF_C1 ± ICF_C2)/(ICF_C3 + ICF_C4).

We then ranked these candidate predictors in each category based on their correlation with ICB response using data from (32). Here, the strength of the correlation was measured by the area under the receiver operating characteristic curve (AUC). The Mann–Whitney U test was used to calculate the *P* values against the null hypothesis of “the AUC is equal to 0.5” (41). AUCs and corresponding *P* values for predicting both the ICB RECIST response (*n* = 16) and the ICB pathologic response (*n* = 26) were calculated. Predictors with *P* < 0.05 for both RECIST response and pathologic response were considered as potential signatures.

### Survival analyses

All Kaplan–Meier analyses were performed by comparing the survival of patients with high signature scores (> median) with those with low scores (≤ median) using a two-sided log-rank test.

### Statistical analyses

For all the multiple comparisons performed, we employed the Benjamini–Hochberg correction method to adjust the *P* values.

### Data availability

In this study, the primary sources of the data are obtained from NCBI Gene Expression Omnibus (GEO; http://www.ncbi.nlm.nih.gov/geo/; RRID: SCR_005012). All data used were sourced from publicly accessible repositories that adhered to ethical standards, including the requirement for written informed consent from patients at the time of initial publication. Additional information about the datasets used in this study can be found in Supplementary Table S1.

All codes that are necessary to reproduce all the results in the article are implemented in Python and R and are publicly available in GitHub: https://github.com/yingstat/TIMEP.

## Results

### The data analyzed and analysis overview

**Figure 1:**
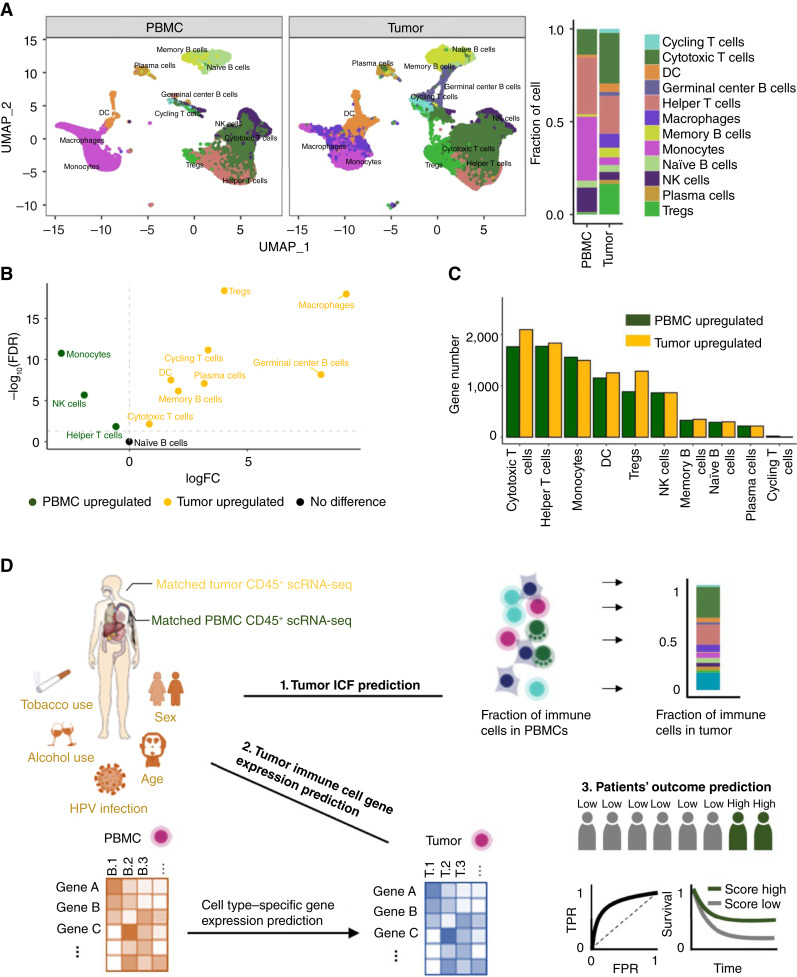
Data basic properties and analysis overview. **A,** Distribution, annotation, and cell fractions of 12 major immune cell types in matched tumor-PBMC CD45^+^ scRNA-seq data from 26 patients with treatment-naïve HNSCC. **B,** A volcano plot showing differential fractions of abundance of 12 major immune cell types between tumor tissues and PBMCs. **C,** Significantly differentially expressed genes in 12 annotated immune cell types from tumor tissues and PBMCs. **D,** An overview of the study: First, **(1)** the ICFs and **(2)** the gene expression levels of major immune cell types in the TME are predicted from the blood scRNA-seq data. Then, **(3)** immune signatures are identified from immune information in the blood and in the TME (which is predicted from the blood) to predict patients’ ICB response. FPR, false-positive rate; TPR, true-positive rate.

We obtained the single-cell transcriptome data from ref. (42), in which all viable CD45^+^ immune cells were measured for matched PBMC samples and primary tumor tissue samples from 18 patients with HPV^−^ and 8 patients with HPV^+^ immunotherapy treatment–naïve HNSCC. Immune cell identities were annotated using *CellTypist*, an automated cell classification tool designed in ref. (35). The resulting cell identities were then merged into 12 major immune cell types such that each cell type in tumor tissues had an average ICF >1% (“Materials and Methods”). ICF is defined as the fraction of cells of a given immune cell type among all CD45^+^ cells in the same sample. Specifically, the 12 immune cell types identified in this study are cycling T cells, cytotoxic T cells, DCs, germinal center B cells, helper T cells, macrophages, memory B cells, monocytes, naïve B cells, NK cells, plasma cells, and Tregs (Fig. 1A). Throughout this study, we quantify cell abundances in term of ICFs.

We first compared the similarity and difference of the immune microenvironment between the TME and the blood of patients with HNSCC. We observed that the ICFs of all immune cell types, except for naïve B cells, are significantly different between tissue samples and matched PBMC samples. Specifically, the ICFs of cycling T cells, cytotoxic T cells, DCs, germinal center B cells, macrophages, memory B cells, plasma cells, and Tregs are higher in the TME, whereas the ICFs of helper T cells, monocytes, and NK cells are lower in the TME (Fig. 1A and B).

When comparing the same immune cell types between the blood and the TME, we found that the expression levels of 100 to 1,000 of genes are significantly altered. Across different immune cell types, there are up to 1,772 genes that are significantly upregulated in PBMC samples and up to 2,097 genes that are significantly upregulated in the tumor (log_2_ FC > 0; FDR < 0.05; Fig. 1C). Specifically, genes that are upregulated in the TME are enriched in specific immune response functions (Supplementary Fig. S1). Our functional analysis revealed that genes upregulated in PBMCs were more enriched in basic housekeeping biological functions such as “rRNA processing” and “cytoplasmic translation,” whereas genes upregulated in TME were more enriched in antitumor immune functions such as “T-cell activation” and “lymphocyte-mediated immunity” (Supplementary Fig. S2).

Next, we evaluated the extent to which we could predict the immune status in the TME from the blood using a machine learning–based framework (Fig. 1D). Briefly, we first predicted the ICFs of 11 of 12 major immune cell types in the TME using the cell types’ ICFs in the matching blood sample and additional clinical information, such as HPV infection status, alcohol and tobacco use, age, and sex. Germinal center B cells were excluded from this analysis as they were found only present in HPV^+^ tumor tissue samples in this study. Second, we predicted the expression level of each gene in each immune cell type in the TME based on its expression level in the corresponding cell type in the matching blood sample along with the patient’s clinical information. Finally, using the immune status information learned from the blood, we identified immune signatures that are predictive of the ICB response of patients with HNSCC.

### ICFs of all cell types in the TME can be predicted from the blood

**Figure 2:**
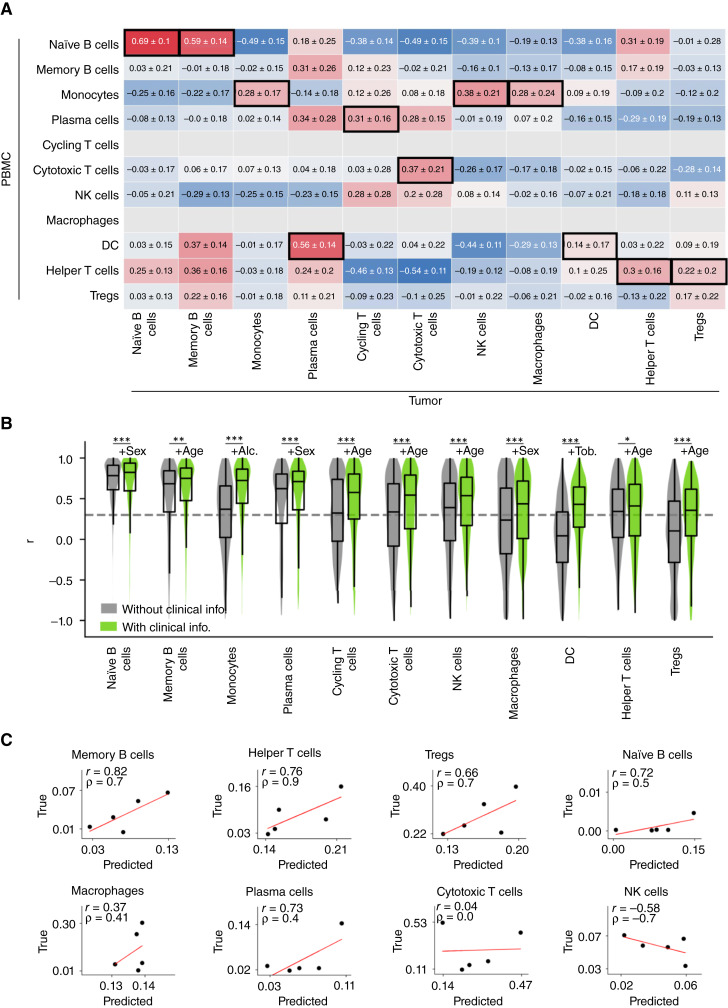
The ICFs of major immune cell types in the TME can be predicted from the blood. **A,** Correlation matrix of ICFs between the TME and the blood. For immune cell types in the TME (columns), the cell types in the blood (rows) with the highest correlations are marked in black boxes. Pearson correlation values shown are mean ± SD over 1,000-replicate bootstrapping. **B,** Distribution of correlations between the model-predicted ICFs and the true TME ICFs are shown on the test sets across 1,000 replicates. **C,** Correlation between the model-predicted TME ICF and the measured TME ICF for each cell type on a small, independent validation dataset (*n* = 5). Cycling T cells are not studied as they are not present in all tumor tissue samples; monocytes and DC are excluded as their pertaining predictive clinical information is missing. *r* denotes the Pearson correlation coefficient, and ρ denotes the Spearman correlation coefficient. Statistical notation: ***, FDR < 0.001; **, FDR < 0.01; *, FDR < 0.05. FDRs are calculated using Benjamini–Hochberg correction. Alc., alcohol; info., information; Tob., tobacco.

We first explored the correlation between ICFs in the TME and those in the blood. To reduce the impact of potential sampling bias, correlations between ICFs were calculated with bootstrapping by randomly resampling the 25 patient samples for 1,000 replications (note that sample 5 was excluded from the 26 patient samples as an outlier; Supplementary Fig. S3). For different cell types in the TME, the highest correlations with cell types in the blood were very different, with the Pearson correlations ranging from 0.14 to 0.69 (Fig. 2A). More intriguingly, for 6 of 11 cell types in the TME, the most correlated cell types in the blood are different from themselves (Fig. 2A). Among these six cell types, two are not present in the blood and hence matching cells could not have been found (cycling T cells and macrophages); two are moderately positively correlated with themselves between the TME and the blood (plasma cells and Tregs); however, the ICFs of two cell types in the blood and in the tumor samples are not correlated at all (memory B cells and NK cells), suggesting that they may play different functional roles in the TME versus in the blood or that their recycling rates between the tumor and the blood are low.

We then aimed to improve the prediction of ICFs in the tumor from the blood ICFs beyond the basic correlations denoted above by building machine learning predictors. Due to the small size of the training data, we focused on building very compact prediction models to minimize the risk of overfitting as much as possible. Specifically, for each cell type in the TME, a one-variable linear regression model was built by using the ICF of its most correlated cell type in the blood (as the only variable), and five two-variable linear regression models were built using the ICF of its most predictive cell type in the blood (as the first variable) and one of the five clinical variables (as the second variable). The most predictive model among those six models was chosen as the final model for predicting that cell type’s ICF. To further overcome overfitting, we randomly split the data into training and test sets (80%: 20%) for 1,000 replicates and performed model training and testing on the 1,000 folds for each cell type (“Materials and Methods”). As shown in Fig. 2B, the ICFs of all 11 immune cell types in the TME were predictable, with mean Pearson correlation coefficients between the predicted and the true ICFs greater than 0.3 (*r* > 0.3), mean normalized mean absolute error less than 1 (NMAE < 1) and a Benjamini–Hochberg adjusted *P* value < 0.05 (FDR < 0.05; Fig. 2B). Notably, clinical information played a statistically significant role in increasing the predictability of the ICFs for all immune cell types, compared with the one-variable baseline models (Fig. 2B).

As these models were generated and tested via a standard cross-validation procedure on the training set, we further tested them (without any further changes) on a smaller, independent, recently published external dataset [*n* = 5; (32)]. Despite the very limited number of matched samples, quite strikingly, six of eight cell types in which ICFs were predictable in the original datasets were also predictable in this test set (with *r* > 0.3). These included the memory B cells, helper T cells, Tregs, naïve B cells, macrophages, and plasma cells. Notably, memory B cells, helper T cells, and Tregs were well predicted with both *r* > 0.6 and ρ (Spearman correlation coefficient) > 0.6 (Fig. 2C).

### The expression levels of 17% to 47% of the genes that are expressed in specific immune cell types in the TME can be predicted from the blood

Similarly, we first examined the correlation between gene expression levels of immune cell types in the TME and those in the blood. As expected, we found that the gene expression levels of all cell types in the TME were most highly correlated with those of the same cell types in the blood, except for cycling T cells and macrophages, which were not found in the blood (Supplementary Fig. S4).

To predict gene expression levels of each immune cell type in the TME, we used a similar model construction strategy. For each gene in each cell type, a one-variable linear regression model was built by using the expression pattern of the gene itself in the corresponding cell type in the blood (as the only variable), and five two-variable linear regression models were constructed using the expression level of the gene itself in the corresponding cell type in the blood (as the first variable) and one of the five clinical variables (as the second variable). The model that had the strongest mean predictive power on the test sets over 1,000 replicates was chosen as the final model (“Materials and Methods”).

**Figure 3:**
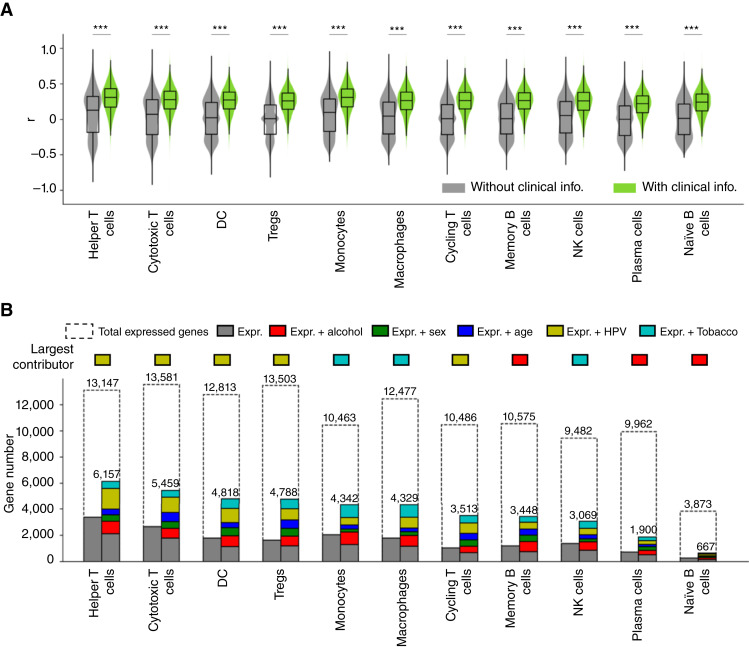
The expression levels of 17% to 47% of the genes expressed in TME immune cells can be predicted from the blood. **A,** Distribution of correlations between the predicted and true expression values of all expressed genes in each immune cell type in the TME, with and without clinical information. **B,** Number of expressed genes and predictable genes in each cell type with and without the consideration of clinical variables. The gray bars represent the number of genes that can be predicted using expression alone, whereas the colored bars indicate the additional number of genes that can be predicted by incorporating clinical variables (see the corresponding colored labels). Statistical notation: ***, FDR < 0.001; **, FDR < 0.01; *, FDR < 0.05. The FDRs are calculated using the Benjamini–Hochberg correction method. info., information.

As observed in the prediction of for ICF, clinical information plays a significant role in the prediction of gene expression as well (Fig. 3A). Notably, various clinical variables seem to have varying levels of significance across different cell types. HPV infection status is the most crucial factor in predicting the expression of DCs and all T cells, tobacco use is the most crucial factor for innate immune cells (i.e., monocytes, macrophages, and NK cells), and alcohol use is the most crucial factor for plasma cells and all B cells (Fig. 3B).

Overall, the expression levels of 17% to 47% of the genes expressed in immune cells in the TME can be predicted from the blood (mean *r* > 0.3 and mean NMAE < 1 with FDR < 0.05 on the test sets over 1,000 replicates; Fig. 3B). Notably, DCs and helper T cells, cytotoxic T cells, and Tregs have the highest number of predictable genes (> 4,700), whereas plasma cells and naïve B cells have the least (< 2,000 and < 700 genes, respectively). The detailed lists of predictable genes in different immune cell types can be found in Supplementary Material SM1. Furthermore, our findings indicate that 22% to 57% of the significantly differentially expressed genes between the TME and the blood (Fig. 1C) are predictable across different immune cell types (Supplementary Fig. S5).

Functional GO enrichment analyses of the predictable genes in different immune cell types in the TME reveals a number of common immune response-related GO terms shared across different immune cell types, such as “adaptive immune response,” “leukocyte cell–cell adhesion,” “positive regulation of T-cell activation,” “response to virus,” “T cell activation,” “antigen processing and presentation,” and “Th17 cell differentiation” (Supplementary Table S2). Notably, genes that are most accurately predicted by the HPV infection status information are enriched in virus infection–related terms. For example, in helper T cells, these genes are enriched in “DNA repair,” “HIV infection,” and “HIV life cycle”; and in Tregs, these genes are enriched in “influenza infection” (Supplementary Fig. S6).

### Exhausted T-cell signature in the TME can be inferred from the blood and the inferred exhausted T-cell score can further predict immunotherapy response in HNSCC

Given the predictability of ICFs and gene expression levels of different immune cell types in the TME, we were interested to test if we could predict the scores of some well-established immune signatures in the TME from the PBMC scRNA-seq data. Specifically, we checked the predictability of three pan-cancer T cell immune signatures for immunotherapy including the exhausted T-cell signature (average expression of PDCD1, CTLA4, LAG3, HAVCR2, and TIGIT in cytotoxic T cells; ref. 43), the cytolytic T-cell signature (average expression of GZMA and PRF1 in cytotoxic T cells; ref. 44), and CXCL13 expression in cytotoxic T cells (45). This investigation was performed by analyzing a dataset of patients with HNSCC (32), including both patient pretreatment blood scRNA-seq data and ICB response information.

**Figure 4:**
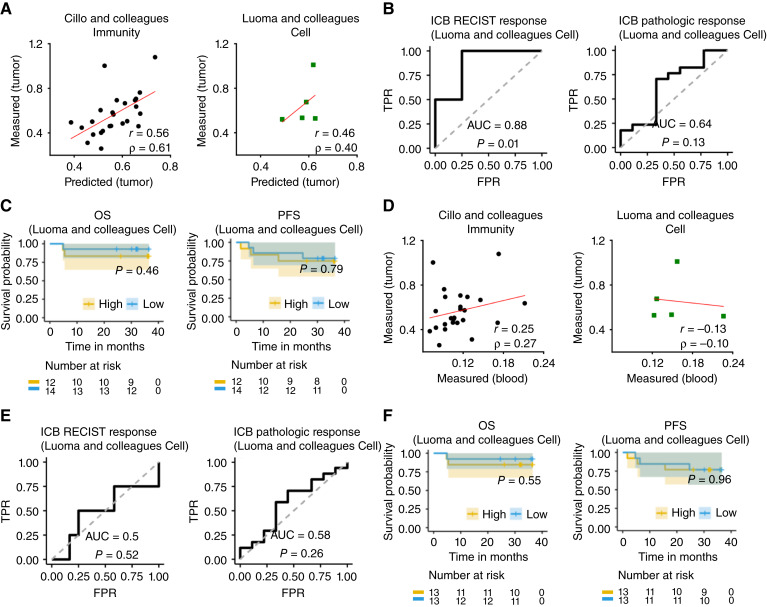
Exhausted T-cell signature in the TME can be inferred from the blood and the inferred exhausted T-cell score can further predict immunotherapy response in HNSCC. **A,** Correlation between the predicted and the measured exhausted T-cell signature scores in TME in the matched blood/tumor training dataset (*n* = 25) and validation dataset (*n* = 5). **B,** ROC curve for predicting ICB RECIST response (*n* = 16) and pathologic response (*n* = 26) by the predicted exhausted T-cell signature scores. **C,** OS and PFS analyses of ICB-treated patients in exhausted-high (exhausted score > quantile 50%) vs. exhausted-low (exhausted score ≤ quantile 50%) tumor groups using the predicted exhausted T-cell signature scores. **D–F** are the same as **A–C**, respectively, except that the predicted exhausted T-cell signature scores in the TME are replaced by the measured exhausted T-cell signature scores in the blood. *r*, Pearson correlation coefficient; ρ, Spearman correlation coefficient. FPR, false-positive rate; TPR, true-positive rate.

First, we found that the exhausted T-cell signature in the TME could be predicted from blood cytotoxic T-cell gene expression levels in both the training dataset of (*r* = 0.56; ρ = 0.61; ref. 42) and the validation dataset of (*r* = 0.46; ρ = 0.40; Fig. 4A; ref. 32). Moreover, the inferred exhausted T-cell signature in the TME was significantly negatively associated with patients’ ICB RECIST response (AUC = 0.88, *P* = 0.01; Fig. 4B), as one would expect. However, the exhausted T-cell signature was not significantly associated with patients’ survival after ICB treatment (Fig. 4C). We also performed Cox proportional hazards regression using the continuously measured exhausted T-cell signature and found that although there were strong trends of HRs > 1, the results were still not statistically significant (OS HR = 11.39, *P* = 0.18; PFS HR = 4.49, *P* = 0.21). A similar trend was found using multivariable Cox proportional hazards regressions by adjusting for age, sex, and HPV infection (OS HR = 3.21, *P* = 0.57; PFS HR = 4.8, *P* = 0.25). This could be attributed to the relatively small sample size of this cohort, and/or the short follow-up time of 36 months, which might not have been sufficient to observe differences in survival among patients, as indicated by the original authors (32). It is worth noting that the exhausted T-cell signature score in the blood on its own is not correlated with that in the TME (Fig. 4D) and is not associated with ICB response or survival (Fig. 4E and F), highlighting the importance of predicting ICB response through the two-step approach. Finally, the cytolytic T-cell and CXCL13 signatures in the TME were less predictable from the blood than the exhaustion signature (Supplementary Fig. S7A and S7D). As a result, the predicted scores for these signatures had low correlations with ICB response and survival (Supplementary Fig. S7B, S7C, S7E, and S7F).

### Prediction of HNSCC immunotherapy response via a newly identified *TME immune signature, ICFR^∗^*

We next examined whether we could identify new blood-based immune signatures that can help predict the ICB response in patients with HNSCC. To this end, we tested two different strategies. Strategy 1 was a direct approach, in which we predicted ICB response based directly on the immune information in the blood. Strategy 2 was an indirect, two-step approach, in which we first identified predictable immune information in the TME from the blood (Figs. 1 and 3) and then used this information to predict ICB response.

**Figure 5:**
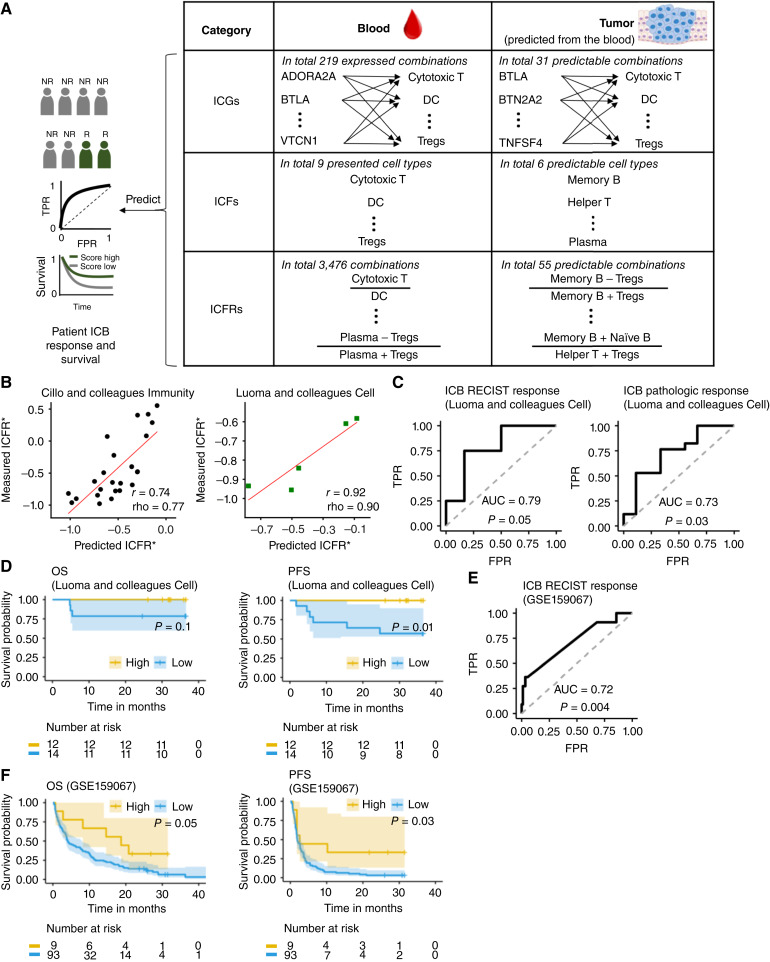
A new blood-predicted tumor immune signature, ICFR*, predicts both response and survival of patients with HNSCC after ICB treatment. **A,** The predictive power for ICB response of three categories of immune information in the blood and in the TME are thoroughly evaluated, including the expression levels of key immune checkpoint genes in their corresponding cell types, ICFs, and ICFRs. **B–F,** Quantifying the prediction accuracy of the ICFR* signature (B_memory_ − Treg)/(B_memory_ + Treg). **B,** Correlation between the predicted ICFR* values and the measured ICFR* values in the TME in the matched blood/tumor training dataset (*n* = 25) and validation dataset (*n* = 5). **C,** ROC curve for predicting ICB RECIST response (*n* = 16) and pathologic response (*n* = 26) by the TME ICFR* signature inferred from the blood scRNA-seq. **D,** OS and PFS analyses of ICB-treated patients in ICFR*-high (score > quantile 50%) vs. ICFR*-low (score ≤ quantile 50%) tumor groups for the PBMC scRNA-seq dataset (GSE200996). **E,** ROC curve for predicting ICB response using the ICFR* computed from the deconvoluted bulk tumor RNA-seq (GSE159067; *n* = 102). **F,** OS and PFS analysis of ICB-treated patients in ICFR*-high vs. ICFR*-low tumor groups for the bulk tumor RNA-seq dataset (GSE159067). *r*, Pearson correlation coefficient; ρ, Spearman correlation coefficient. FPR, false-positive rate; TPR, true-positive rate.

To achieve our goal, we comprehensively examined three categories of immune information in both the blood (for “strategy 1” candidates) and the corresponding predictable immune information in the TME (for “strategy 2” candidates). Specifically, we examined (i) the expression of ICGs, (ii) the levels of OSICFs, and (iii) ICFRs, as the predictive features for ICB response (Fig. 5A). More specifically, in the first category, we studied the expression of 79 ICGs in specific immune cell types, as reported in the literature (“Materials and Methods”). As a given ICG might be expressed in a few cell types, this resulted in a space of 219 gene–cell type pairs, of which the expression state of 31 pairs in the TME could be predicted from the blood. In the second category, we focused on nine ICFs in the blood in which their levels were >0 in the direct approach, and six ICFs in the TME in which their levels could be predicted from the blood for the indirect, two-step approach. In the third category, we comprehensively studied ICFRs in the form of (ICF_C1 ± ICF_C2)/(ICF_C3 + ICF_C4), in which C1, C2, C3, and C4 are immune cell types. This space was composed of 3,476 different ICFRs in the blood, of which 55 ICFRs in the TME could be predicted from the blood. See “Materials and Methods” for more details and see Supplementary Material SM2 for a comprehensive list of items in each category.

We next evaluated the correlation of these three categories of candidates and patients’ ICB response in the HNSCC dataset of ref. (32). Here, the strength of the correlation was measured by AUC. The statistical significance of the correlation was calculated against the null hypothesis of “the AUC is equal to 0.5.” In category (1), we identified a few ICGs, and their expression levels in the blood or in the TME were significantly associated with both ICB RECIST response (*n* = 16) and ICB pathological response (*n* = 26; “Materials and Methods”). The potential ICG signatures in the blood included LGALS9 expression in memory B cells, ICOSLG expression in naïve B cells, CD80 expression in monocytes, CD47 expression in Tregs, and CD96 expression in memory B cells (Supplementary Fig. S8). The potential ICG signatures in the TME, in which their expression levels were predicted from the blood, included LAG3 expression in NK cells, and ICOSLG expression in naïve B cells (Supplementary Fig. S9). In category (2), none of the ICFs, either from the blood or from the TME, was significantly associated with ICB response (Supplementary Material SM2).

We hence focused on category (3), in which there were three ICFR signatures in the TME that were highly predictive (Supplementary Material SM2). The top predictive ICFR signature in the TME was (B_memory_ − Treg)/(B_memory_ + Treg), denoted as ICFR^∗^. First, ICFR^∗ ^values in the TME could be well predicted from the blood, with strong correlations between the predicted values and the measured values in both the training dataset of (*r* = 0.75; ρ = 0.77; ref. 42) and the independent validation dataset of (*r* = 0.96; ρ = 0.90; Fig. 5B; ref. 32). Second, inferred ICFR^∗ ^values in the TME, which were predicted from the blood, were significantly associated with patients’ ICB response (positively correlated with ICB response; RECIST AUC = 0.79, *P* = 0.05; pathological AUC = 0.73, *P* = 0.03; Fig. 5C). Notably, as was the case with the exhausted T-cell signature, the ICFR^∗ ^scores estimated directly from the blood were not significantly associated with ICB response (RECIST AUC = 0.54, *P* = 0.43; pathological AUC = 0.62, *P* = 0.17; Supplementary Fig. S10), in contrast to those inferred in the TME from the blood. Remarkably, none of the 3,476 ICFRs in the blood was significantly associated with ICB response (Supplementary Material SM2), which further underscored the importance of predicting ICB response via the two-step approach. Finally, although signatures from category (1) were not significantly associated with patients’ survival after ICB treatment (Supplementary Figs. S8 and S9), which might be due to the short follow-up time issue of this dataset as mentioned above, the ICFR^∗ ^scores in the TME were predictive of patients’ survival after ICB treatment (OS, *P* = 0.1; PFS, *P* = 0.01; Fig. 5D).

To further test the robustness of the ICFR^∗ ^signature in predicting ICB response, we analyzed an additional large cohort. This cohort was a recently released dataset (46) consisting of bulk tumor RNA-seq of 102 patients with advanced HNSCC treated with ICB. Although it did not include matched single-cell data, we were still able to use it to test the predictive power of the ICFR^∗ ^signature, as described below. To calculate the ICFR^∗ ^values in the TME in each of these tumor samples, we inferred the relative abundance of each immune cell type by deconvolving the bulk tumor RNA-seq data via CIBERSORT (47). Remarkably, the ICFR^∗ ^scores were also predictive of ICB response in this independent dataset (AUC = 0.72, *P* = 0.004; Fig. 5E), which was much higher than the predictive power obtained using the FDA-approved TMB biomarker in HNSCC [AUC = 0.56, *P* = 0.74; *n* = 69; Supplementary Fig. S11; data from the MSK-IMPACT cohort (48)]. Additionally, ICFR^∗ ^values significantly predicted patients’ survival after ICB treatment (OS, *P* = 0.05; PFS, *P* = 0.03; Fig. 5F). It was also worth noting that ICFR^∗ ^did not predict survival of patients with HNSCC in The Cancer Genome Atlas (TCGA), in which patients were not treated with ICB (Supplementary Fig. S12), demonstrating its specificity for predicting ICB response.

For completeness, we also evaluated the predictive power of the two other ICFR signatures that ranked high on our list. One was (B_memory_ − Treg)/(Treg + Plasma), which was positively correlated with ICB response (RECIST AUC = 0.79, *P* = 0.05; pathological AUC = 0.73, *P* = 0.03; Supplementary Fig. S13). The other was B_memory_/(Treg + Plasma), which was also positively correlated with ICB response (RECIST AUC = 0.79, *P* = 0.05; pathological AUC = 0.73, *P* = 0.03; Supplementary Fig. S13). Notably, these two ICFR signatures were also able to significantly predict the ICB response in the bulk dataset (AUC = 0.72, *P* = 0.004 and AUC = 0.63, *P* = 0.007, respectively) but were less predictive of patients’ survival after ICB treatment (Supplementary Fig. S13).

## Discussion

Cancer is a systemic disease that affects the entire body, altering the composition and function of the immune system as a whole (49). Charting the immune status of the TME in individuals is crucial for understanding cancer progression, drug response, and resistance, which can help promote precision medicine (50). Due to the challenges and risks associated with tumor tissue biopsies, characterizing the TME from liquid biopsies has become one of the most important directions in cancer prognosis, stratification, and monitoring (51). Leveraging the advantage of single-cell sequencing techniques, here, we have established a machine learning framework utilizing the global expression profiles of major immune cells in the peripheral blood, derived from the CD45^+^ scRNA-seq data, to infer the immune status of the TME in patients with HNSCC (Fig. 1D). To the best of our knowledge, the immune status of the TME has not yet been comprehensively predicted from the blood previously.

Our results testify that ICFs of major immune cell types in the TME of HNSCC primary tumors can be inferred from the blood. The most predictable three immune cell types are memory B cells, Tregs, and helper T cells on the independent validation data (Fig. 2C). Coincidently, all these cell types have been shown to play important roles in regulating cancer prognosis and drug resistance in patients with HNSCC (52, 53). Additionally, we show that the expression levels of approximately 20% to 50% of the genes expressed in different immune cell types in the TME is strongly correlated between the blood and the TME in (mostly) the same cell types and can be predicted from the blood. Furthermore, our findings suggest that the clinical information significantly contributes to the predictability of both ICFs and gene expression levels of immune cells in the TME. Interestingly, different clinical variables are found to contribute to the prediction of the expression profiles of different immune cells. For example, among all five clinical variables, HPV infection status is found to predict the expression of the largest number of genes in DCs and all T cells, whereas tobacco use information is the largest contributor for innate immune cells (i.e., monocytes, macrophages, and NK cells), and alcohol use is the largest contributor for B cells and plasma cells (Fig. 3B). This suggests that different clinical variables may have different impacts on the gene expression levels of different cell types.

Turning to study the ability to predict patients’ response to ICB from the inferred ICFs and gene expression in the TME, we show that one of the most well-known pan-cancer immune signatures for immunotherapy, the exhausted T-cell signature, can be predicted from the blood single-cell transcriptomics in HNSCC and the inferred exhausted T-cell signature from the blood can predict patients’ ICB response (Fig. 4A and B). Moreover, we identify a new immune signature, ICFR^∗^, and its inferred TME scores from the blood are more strongly associated with patients response. Specifically, the ICFR^∗^ predicts both ICB response and survival on both single-cell and bulk datasets (Fig. 5B–F). Additionally, it is worth noting that the predictive power of ICFR^∗^ holds across different patient populations: the single-cell cohort contains only patients with newly diagnosed oral cavity squamous cell carcinoma, who have received the ICB as a neoadjuvant therapy before the surgery, whereas in the bulk cohort, the ICB treatment has been used as a main therapy for patients with advanced HNSCC and more than half of the patients have received at least one line therapy before the ICB treatment. In addition, ICFR^∗^ is not predictive of the survival of patients with HNSCC that are not treated with ICB therapy, in the TCGA cohort. Taken together, these results demonstrate that the prediction ability of ICFR^∗^ is fairly robust and also specific to immunotherapy treatment. It is also worth noting that in contrast to the predictive power of inferred ICFR^∗^ scores in the TME, none of the totaling ˜3,500 candidate ICFR combinations in the blood are directly predictive of ICB response, which highlights the importance of constructing a two-step predictor to predict ICB treatment outcome, i.e., first learning about the immune status of the TME from the blood data, and then using the latter to further predict the treatment outcome. Notably, we find that B cells play a crucial role in the three top ICFR signatures identified in this research, which is in line with recent studies that demonstrate the importance of B cells on ICB treatment outcome (54–56).

The present study has some limitations that should be acknowledged. First, the sample size used to train the models is relatively small, as the study relies on yet costly scRNA-seq data. This limitation makes it challenging to perform stratified cross-validation or to train and validate the models in separate cohorts for important factors like HPV infection status. Currently, there is no larger publicly available dataset that contains matched tumor-PBMC scRNA-seq data. Although by constructing very compact predictors, the risk of overfitting has been reduced and the resulting models have shown reasonable performance on an independent validation dataset, larger cohorts are still needed to further test and validate the results. Specifically, as the predictive power of inferred TME ICFR^∗ ^signature has only been tested in one single-cell dataset, more PBMC cohorts are needed to further test and validate this potentially clinically relevant biomarker. Second, the focus of this study is on the relative fractions of immune cells, as the fractions of different immune cells of all cells (including malignant cells and other hematopoietic cells) are not available in current datasets. Such future studies may potentially further enhance ICB response prediction. Third, we observe an interesting correlation pattern between ICFs in the TME and blood (Fig. 2A), which suggests that there may be possible interactions between immune cells in the TME and blood that may have been previously overlooked. To predict ICFs for a specific immune cell type in the TME, we used the most highly correlated cell type in the blood to build our predictive models as a starting point. This can be conceived as a prior feature selection step that may introduce a potential bias when evaluating the model in cross-validation. However, given the limited sample size, the current strategy is the best option one could adopt. If we would have instead tested the ICFs of all cell types and all the clinical variables, the number of candidate models would have greatly exceeded the training sample size, making that analysis implausible. It is worth mentioning that it was reported that CD4^+^ and CD8^+^ T-cell levels in paired tumor and blood samples were inversely correlated in patients with melanoma (57). However, this was not the case in HNSCC (cytotoxic T cells, *r* = 0.37; helper T cells, *r* = 0.3; Tregs, *r* = 0.17; Fig. 2A). This discrepancy indicates that there may be different cell transit patterns between tumor and blood in different cancer indications. Fourth, the transcriptomic sequencing data used in this study remains costly and are not commonly included in routine clinical practices. However, its expense can be seen as justifiable when considering the average annual cancer treatment cost in the United States, which is approximately $150,000. The additional cost of sequencing patient tissue samples may be a worthwhile investment in enhancing treatment precision and outcomes (58, 59). Finally, in the future, when more data become available, it will be important to revisit and investigate the robustness and underlying mechanisms of the relationship between ICFs in the TME and blood in HNSCC and more other cancer indications. With future increased sample size, one could then also use more robust cross-validation models to test and validate the prediction results in more depth.

In summary, transcriptome-based biomarkers are showing increasing power in predicting cancer prognosis and immunotherapy response. Here, we have shown the feasibility of constructing predictive models of immune cell fractions and gene expression levels in the TME based on matching blood single-cell transcriptomics. We further show that the latter TME blood-derived information is in turn predictive of patients’ ICB response. These results offer a new and promising way to extend the realm of liquid biopsies beyond ctDNAs and CTCs to that of PBMC transcriptomics. Taken together, our work represents a promising initial step toward utilizing single-cell transcriptomics based liquid biopsies to accurately predict the immune status of patients’ tumors from their blood, facilitating personalized cancer precision therapy in the future.

## Supplementary Material

Supplementary Material SM2 Comprehensive lists of all signatures predicting patient ICB response.

Supplementary Figure 1 Supplementary Figure 1

Supplementary Figure 2 Supplementary Figure 2

Supplementary Figure 3 Supplementary Figure 3

Supplementary Figure 4 Supplementary Figure 4

Supplementary Figure 5 Supplementary Figure 5

Supplementary Figure 6 Supplementary Figure 6

Supplementary Figure 7 Supplementary Figure 7

Supplementary Figure 8 Supplementary Figure 8

Supplementary Figure 9 Supplementary Figure 9

Supplementary Figure 10 Supplementary Figure 10

Supplementary Figure 11 Supplementary Figure 11

Supplementary Figure 12 Supplementary Figure 12

Supplementary Figure 13 Supplementary Figure 13

Supplementary Table 1 Supplementary Table 1

Supplementary Table 2 Supplementary Table 2

Supplementary Material SM1 A list of predictable genes in different immune cell types.
