# Supplementary material for: Inferring Characteristics of the Tumor Immune Microenvironment of Patients with HNSCC from Single-Cell Transcriptomics of Peripheral Blood
Source: Cancer Res Commun. 2024 Sep 5;4(9):2335–48. doi: 10.1158/2767-9764.CRC-24-0092 (PMC11375407; doi:10.1158/2767-9764.CRC-24-0092)
Supplement: Supplementary Figure 1 [file crc-24-0092_supplementary_figure_1_suppsf1.pdf]

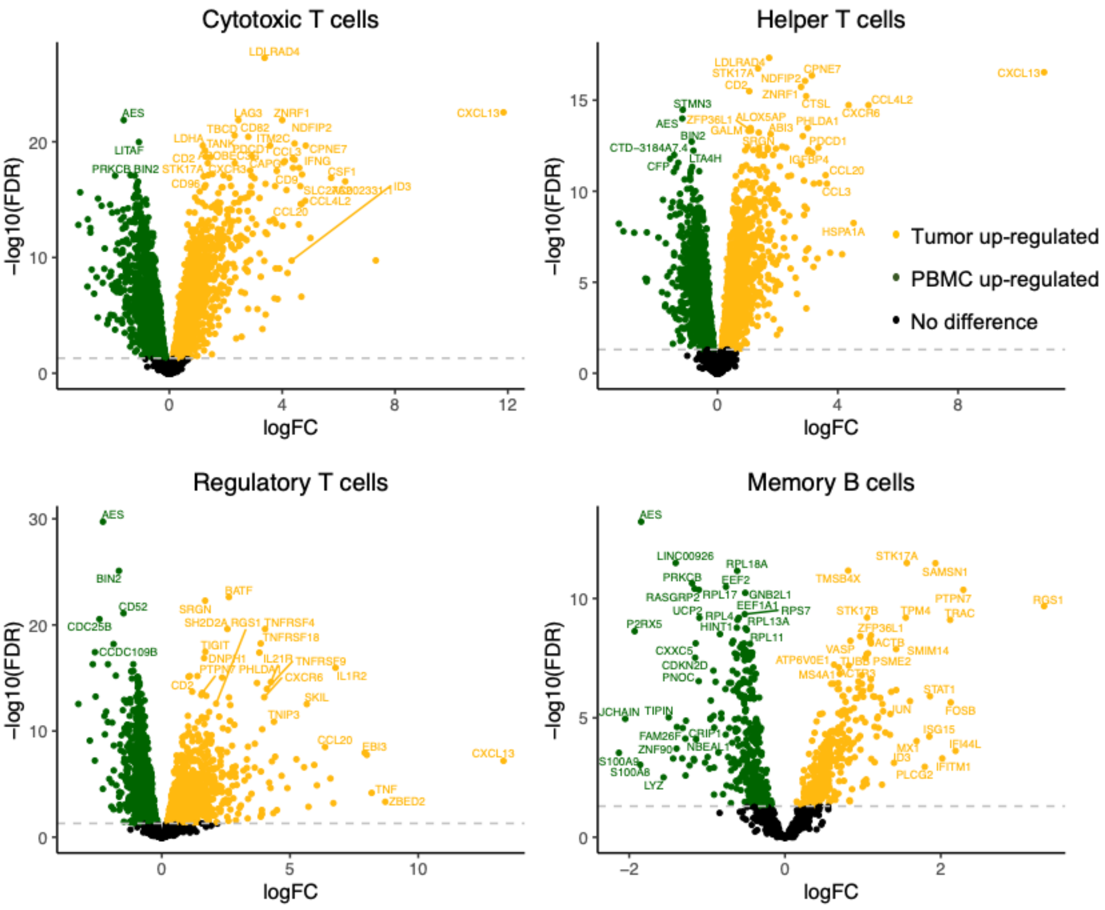

Supplementary Figure 1. Immune response related genes are more highly expressed in the TME compared to the blood.
