# Supplementary material for: Inferring Characteristics of the Tumor Immune Microenvironment of Patients with HNSCC from Single-Cell Transcriptomics of Peripheral Blood
Source: Cancer Res Commun. 2024 Sep 5;4(9):2335–48. doi: 10.1158/2767-9764.CRC-24-0092 (PMC11375407; doi:10.1158/2767-9764.CRC-24-0092)
Supplement: Supplementary Figure 2 [file crc-24-0092_supplementary_figure_2_suppsf2.pdf]

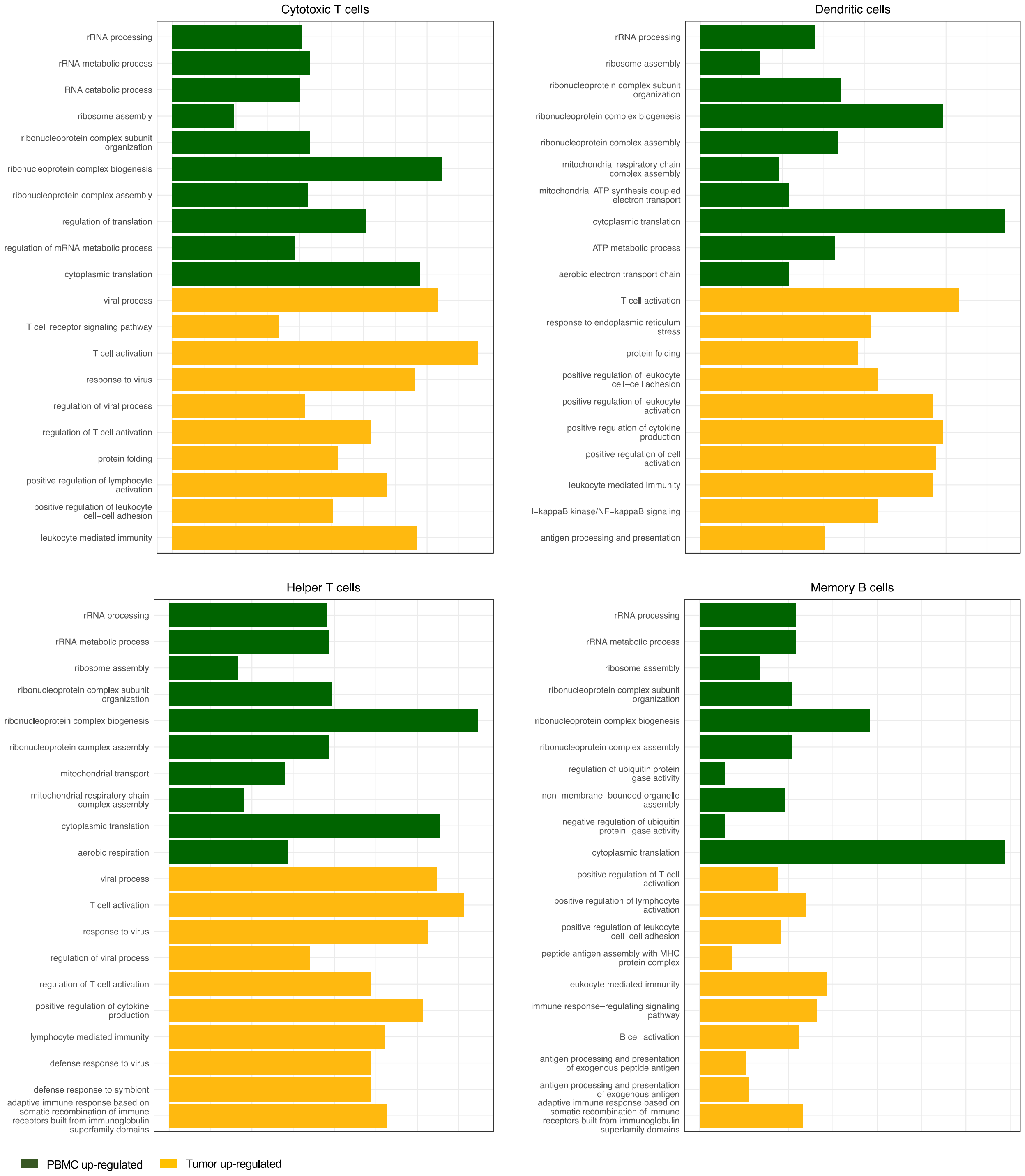

Supplementary Figure 2 (continued next page)

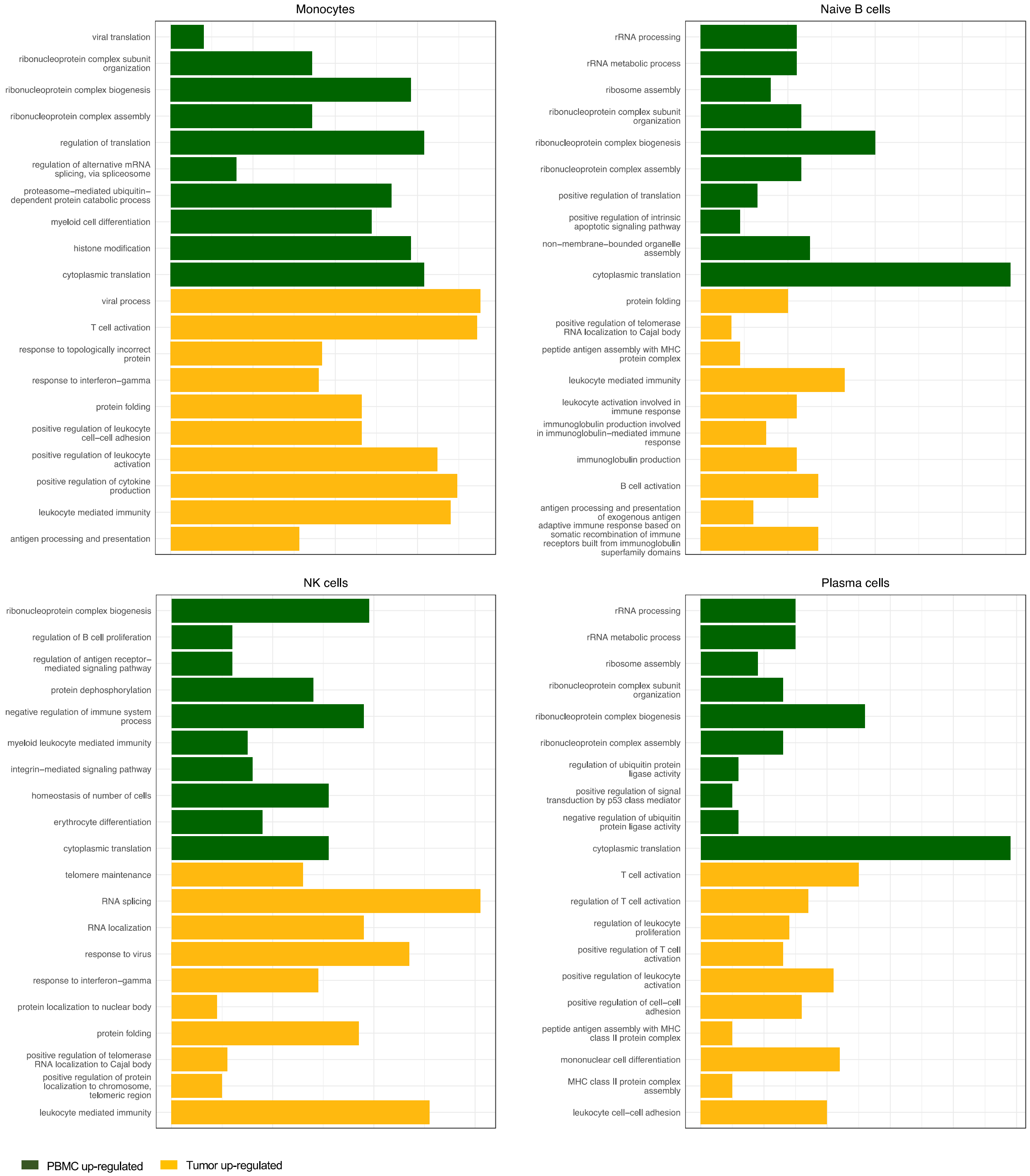

Supplementary Figure 2 (continued next page)

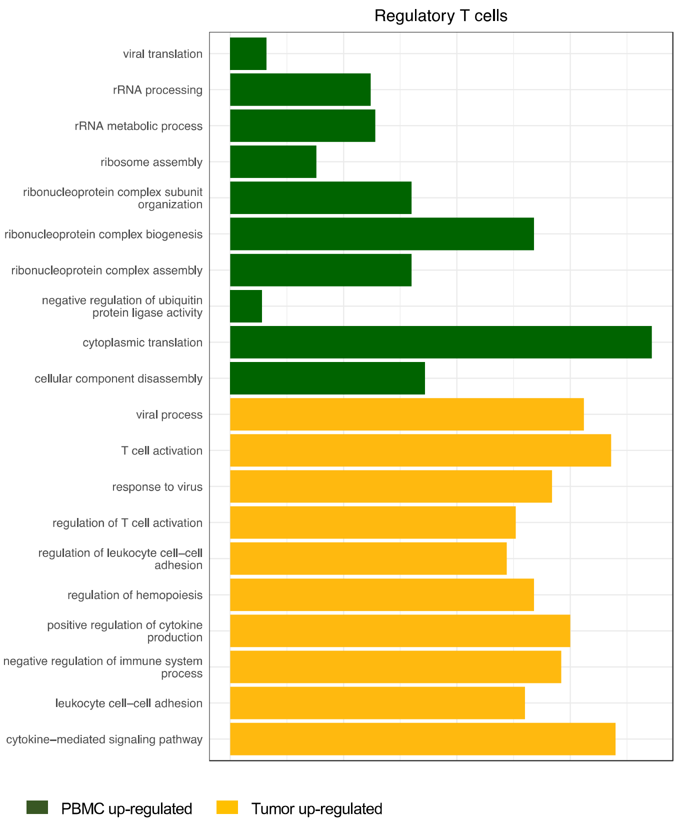

Supplementary Figure 2. Functional enrichment of up-regulated genes in different immune cells in the TME and in the blood.
