# Supplementary material for: Inferring Characteristics of the Tumor Immune Microenvironment of Patients with HNSCC from Single-Cell Transcriptomics of Peripheral Blood
Source: Cancer Res Commun. 2024 Sep 5;4(9):2335–48. doi: 10.1158/2767-9764.CRC-24-0092 (PMC11375407; doi:10.1158/2767-9764.CRC-24-0092)
Supplement: Supplementary Figure 3 [file crc-24-0092_supplementary_figure_3_suppsf3.pdf]

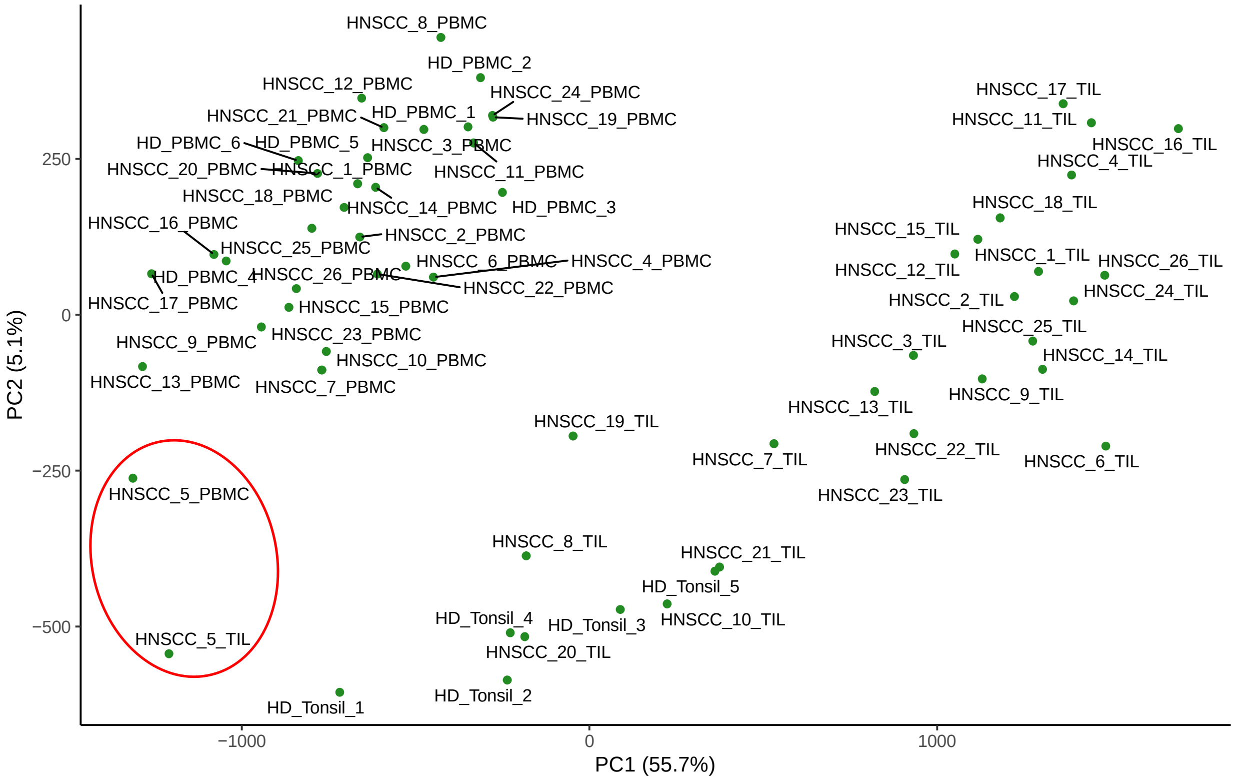

Supplementary Figure 3. A PCA plot of the pseudo-bulk gene expression data from CD45+ PBMC and primary tumor tissue samples from 26 HNSCC patients and 11 healthy donors. Sample 5 from the HNSCC patients is excluded from further analysis as an outlier, which means that it is significantly different from the other samples and may not be representative of the group.
