# Supplementary material for: Inferring Characteristics of the Tumor Immune Microenvironment of Patients with HNSCC from Single-Cell Transcriptomics of Peripheral Blood
Source: Cancer Res Commun. 2024 Sep 5;4(9):2335–48. doi: 10.1158/2767-9764.CRC-24-0092 (PMC11375407; doi:10.1158/2767-9764.CRC-24-0092)
Supplement: Supplementary Figure 4 [file crc-24-0092_supplementary_figure_4_suppsf4.pdf]

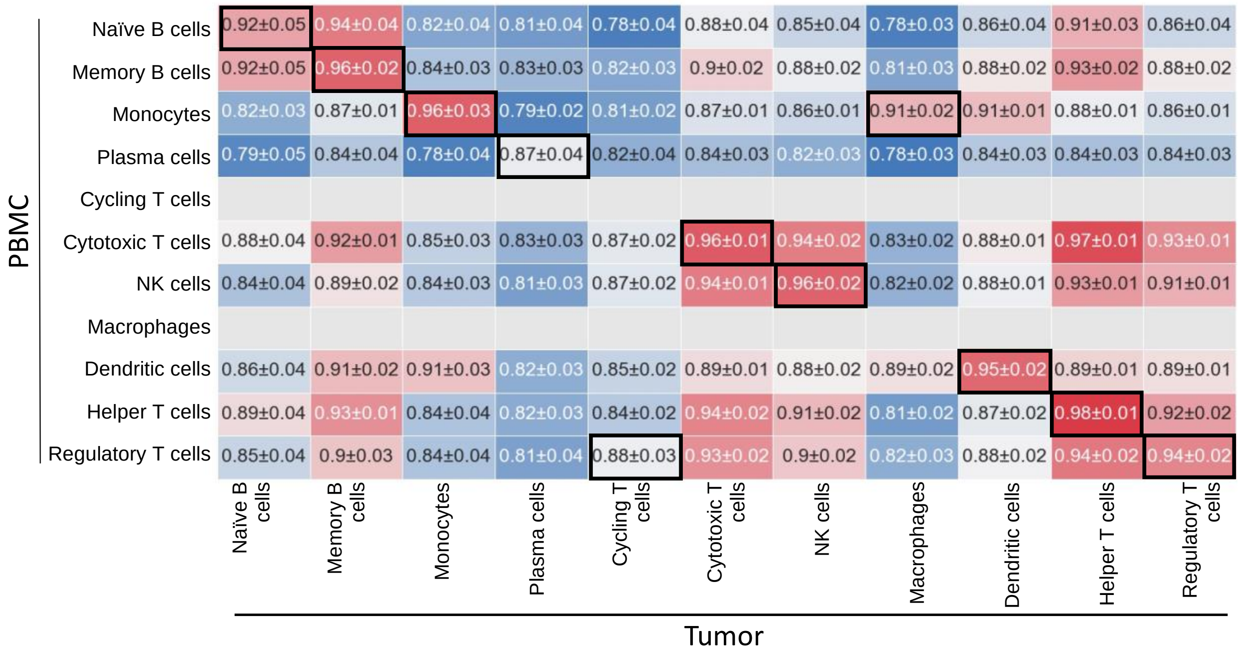

Supplementary Figure 4. Correlation matrix that compares whole-genome gene expression levels of major immune cell types in the TME to those in the blood. The cells in the TME are listed as columns and the cells in the blood are listed as rows. The matrix shows the correlation between the overall gene expression levels of each cell type in the TME and the corresponding cell type in the blood. The cell type in the blood that best correlates with a given cell type in the TME is marked in a black box. The correlation is calculated by flattening the expression levels of all genes in a cell type (mean ± s.d., where s.d. are calculated across the 25 samples).
