# Supplementary material for: Inferring Characteristics of the Tumor Immune Microenvironment of Patients with HNSCC from Single-Cell Transcriptomics of Peripheral Blood
Source: Cancer Res Commun. 2024 Sep 5;4(9):2335–48. doi: 10.1158/2767-9764.CRC-24-0092 (PMC11375407; doi:10.1158/2767-9764.CRC-24-0092)
Supplement: Supplementary Figure 5 [file crc-24-0092_supplementary_figure_5_suppsf5.pdf]

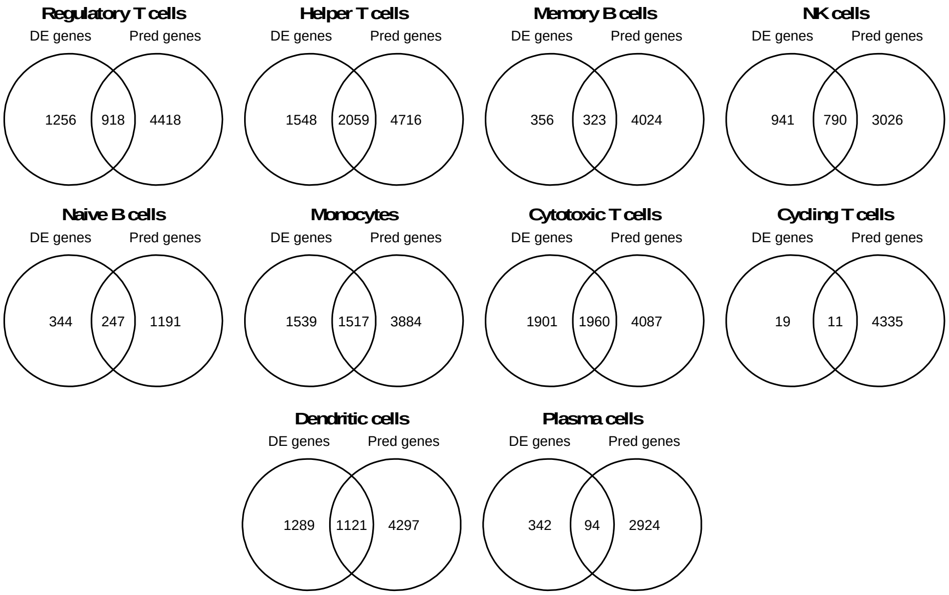

Supplementary Figure 5. The expression levels of 22-57% differentially expressed genes between immune cells in the TME and in the blood can be predicted from the blood. Note: DE genes, differentially expressed genes in immune cells between the TME and the blood; Pred genes, genes whose expression levels in immune cells in the TME can be predicted from the blood.
