# Supplementary material for: Inferring Characteristics of the Tumor Immune Microenvironment of Patients with HNSCC from Single-Cell Transcriptomics of Peripheral Blood
Source: Cancer Res Commun. 2024 Sep 5;4(9):2335–48. doi: 10.1158/2767-9764.CRC-24-0092 (PMC11375407; doi:10.1158/2767-9764.CRC-24-0092)
Supplement: Supplementary Figure 6 [file crc-24-0092_supplementary_figure_6_suppsf6.pdf]

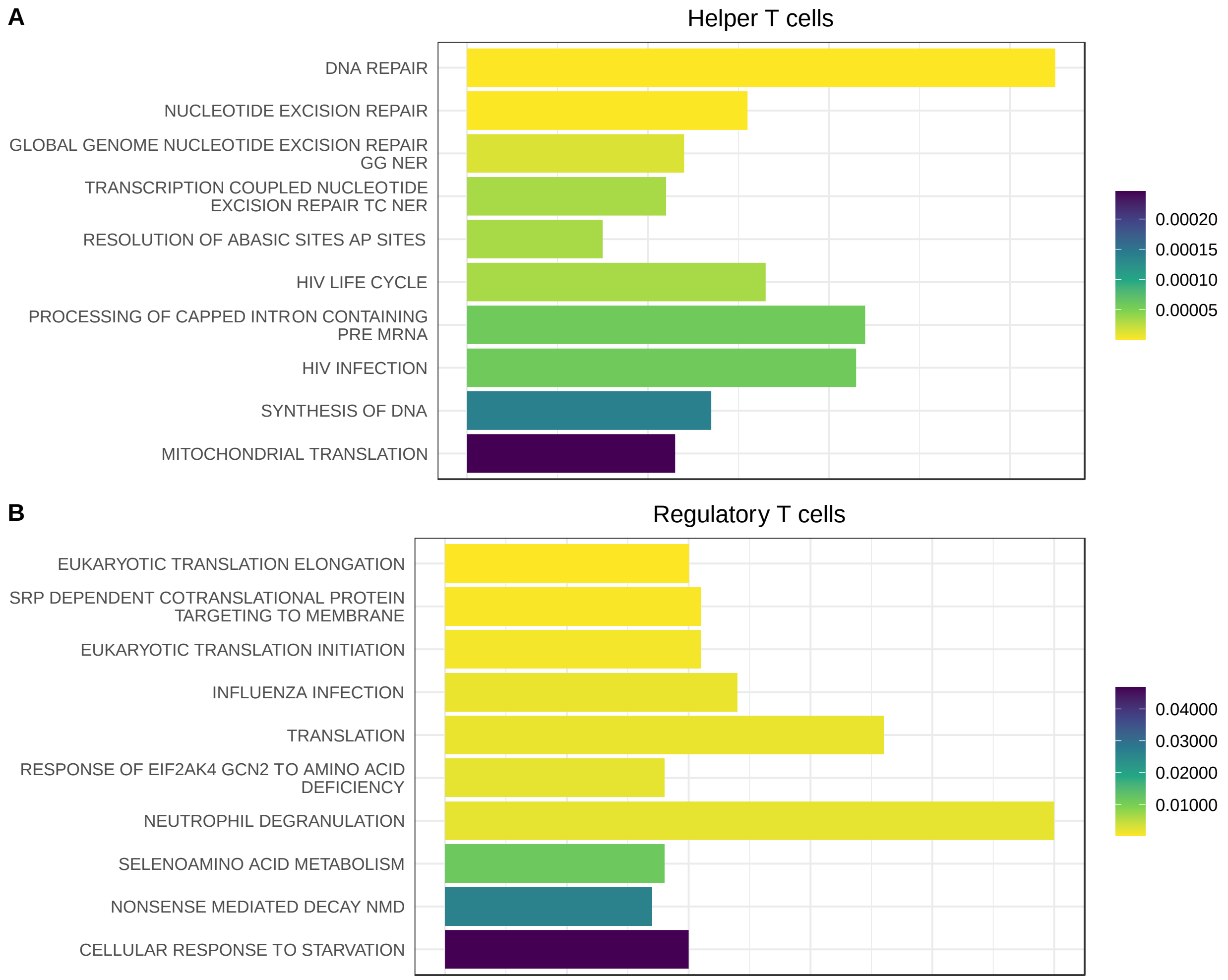

Supplementary Figure 6. Reactome functional enrichment of genes in helper T cells (A) and regulatory T cells (B) that are most accurately predicted based on the HPV infection status information.
