# Supplementary material for: Inferring Characteristics of the Tumor Immune Microenvironment of Patients with HNSCC from Single-Cell Transcriptomics of Peripheral Blood
Source: Cancer Res Commun. 2024 Sep 5;4(9):2335–48. doi: 10.1158/2767-9764.CRC-24-0092 (PMC11375407; doi:10.1158/2767-9764.CRC-24-0092)
Supplement: Supplementary Figure 7 [file crc-24-0092_supplementary_figure_7_suppsf7.pdf]

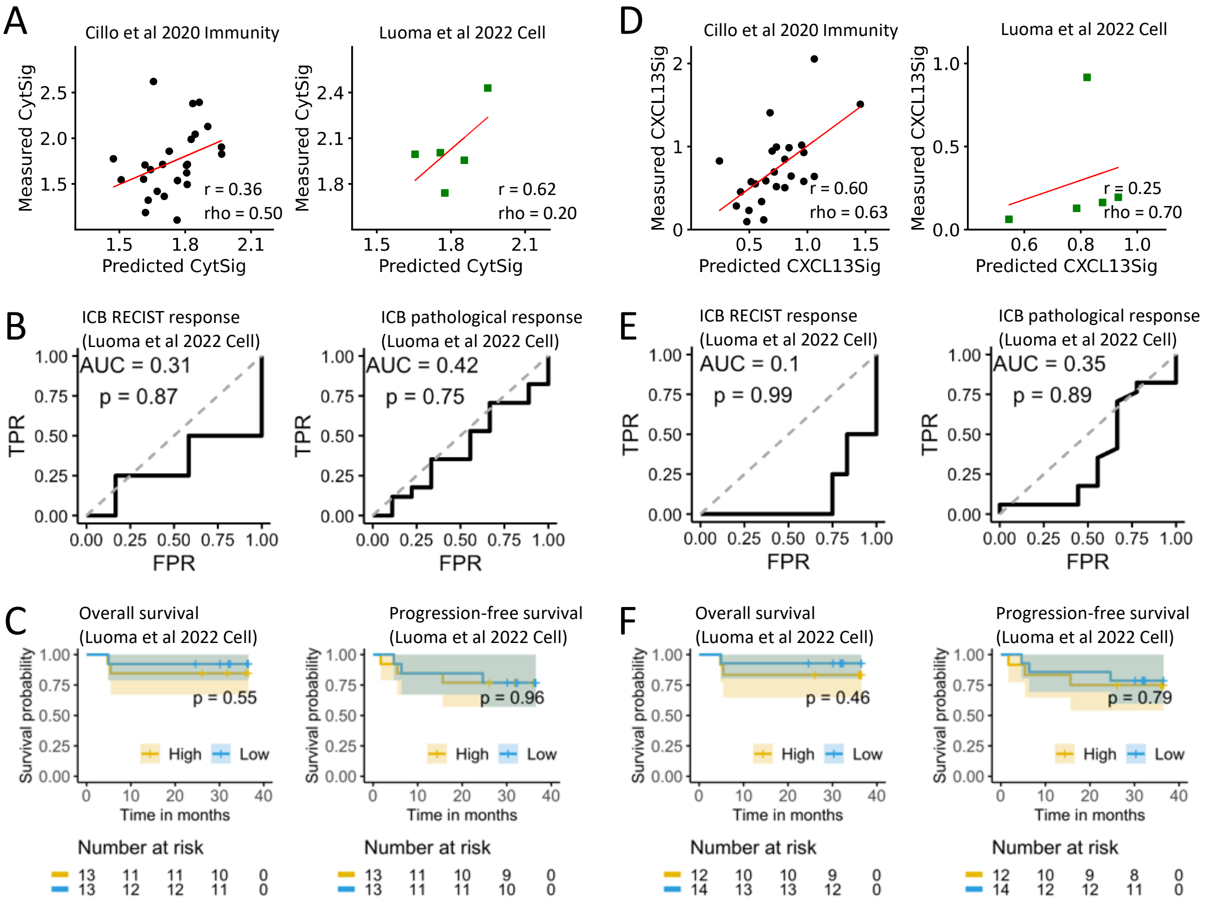

Supplementary Figure 7. The cytolytic T-cell and the CXCL13 signatures in the TME are less predictable from the blood. A. Correlation between the predicted and the measured cytolytic T-cell signature scores in TME in the matched blood/tumor training dataset (n = 25) and validation dataset (n = 5). **B**. ROC curve for predicting ICB RECIST response (n = 16) and pathological response (n = 26) by the predicted cytolytic T-cell signature scores. **C.** Overall survival and progression-free survival analyses of ICB-treated patients in cytolytic-high (cytolytic score > quantile 50%) versus cytolytic-low (cytolytic score ≤ quantile 50%) tumor groups using the predicted cytolytic T-cell signature scores. Panels **D**, **E**, **F** are the same to A, B, C, respectively, except that the cytolytic T-cell signature is replaced by the CXCL13 signature. Abbreviations: r, Pearson correlation coefficient; rho, Spearman correlation coefficient; TPR, true positive rate; FPR, false positive rate.
