# Supplementary material for: Inferring Characteristics of the Tumor Immune Microenvironment of Patients with HNSCC from Single-Cell Transcriptomics of Peripheral Blood
Source: Cancer Res Commun. 2024 Sep 5;4(9):2335–48. doi: 10.1158/2767-9764.CRC-24-0092 (PMC11375407; doi:10.1158/2767-9764.CRC-24-0092)
Supplement: Supplementary Figure 9 [file crc-24-0092_supplementary_figure_9_suppsf9.pdf]

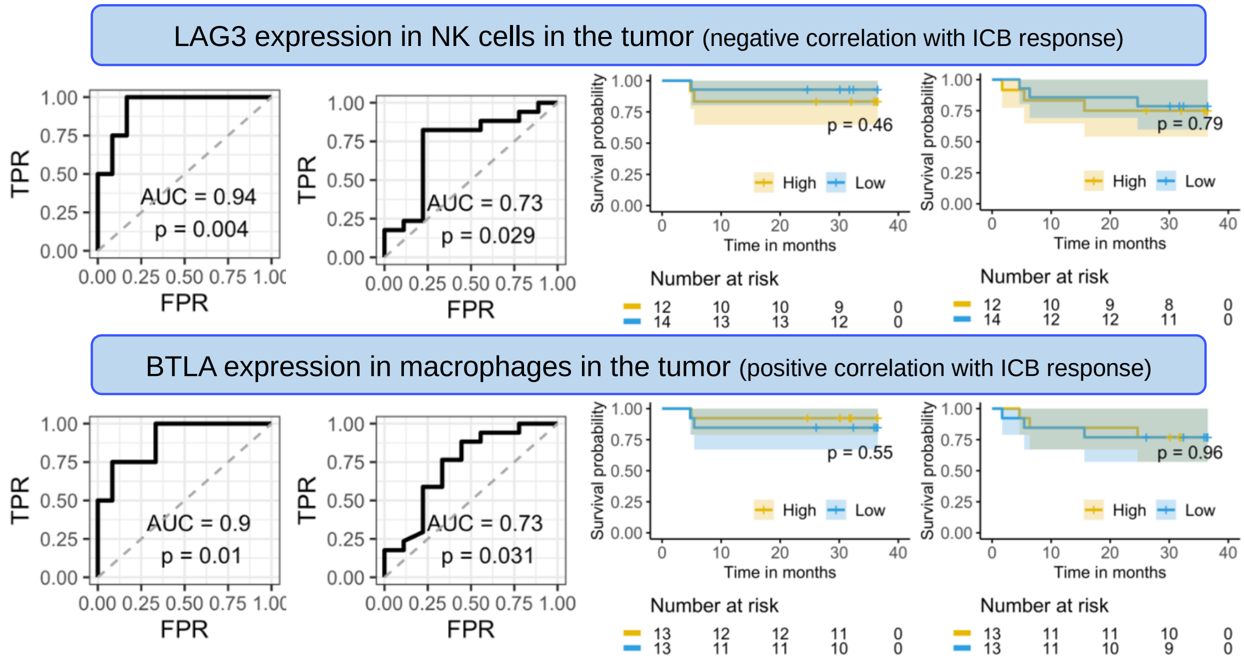

Supplementary Figure 9. Two tumor ICG signatures are found to predict ICB response but patient survival after ICB treatment in HNSCC patients. The names of the two signatures are shown in the figure. The first two columns are ROC curves of ICB RECIST response and pathological response respectively. The last two columns are overall survival and progression-free survival after ICB treatment respectively. Note that the gene expression levels in the TME are predicted from the blood using the machine learning models.
