# Supplementary material for: Inferring Characteristics of the Tumor Immune Microenvironment of Patients with HNSCC from Single-Cell Transcriptomics of Peripheral Blood
Source: Cancer Res Commun. 2024 Sep 5;4(9):2335–48. doi: 10.1158/2767-9764.CRC-24-0092 (PMC11375407; doi:10.1158/2767-9764.CRC-24-0092)
Supplement: Supplementary Figure 11 [file crc-24-0092_supplementary_figure_11_suppsf11.pdf]

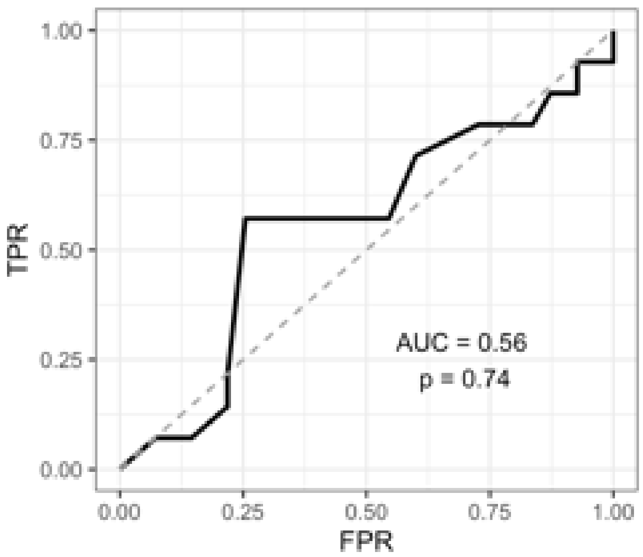

Supplementary Figure 11. The predictive power of the TMB for ICB response in HNSCC patients in the MSK-IMPACT cohort. This cohort consists of in total 69 patients with 14 of them being responders.
