# Supplementary material for: Inferring Characteristics of the Tumor Immune Microenvironment of Patients with HNSCC from Single-Cell Transcriptomics of Peripheral Blood
Source: Cancer Res Commun. 2024 Sep 5;4(9):2335–48. doi: 10.1158/2767-9764.CRC-24-0092 (PMC11375407; doi:10.1158/2767-9764.CRC-24-0092)
Supplement: Supplementary Figure 12 [file crc-24-0092_supplementary_figure_12_suppsf12.pdf]

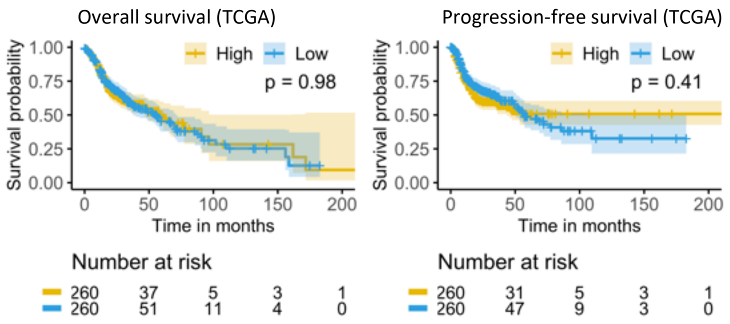

Supplementary Figure 12. The tumor ICFR signature of (B_{memory} - T_{reg}) / (B_{memory} + T_{reg}) is not found to predict the survival of HNSCC patients in the TCGA dataset, where patients were not treated with ICB.
