# Supplementary material for: Inferring Characteristics of the Tumor Immune Microenvironment of Patients with HNSCC from Single-Cell Transcriptomics of Peripheral Blood
Source: Cancer Res Commun. 2024 Sep 5;4(9):2335–48. doi: 10.1158/2767-9764.CRC-24-0092 (PMC11375407; doi:10.1158/2767-9764.CRC-24-0092)
Supplement: Supplementary Figure 13 [file crc-24-0092_supplementary_figure_13_suppsf13.pdf]

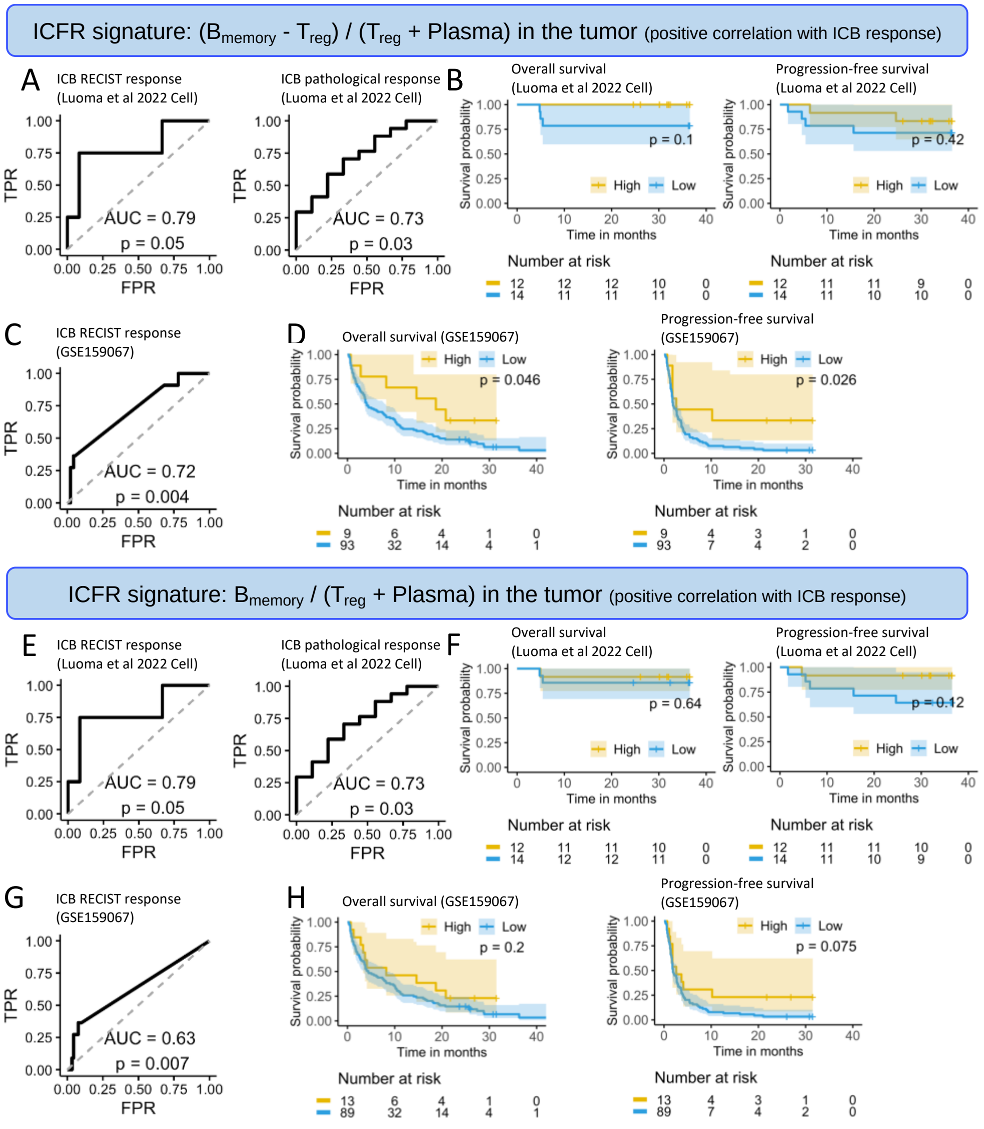

Supplementary Figure 13. **Two additional tumor ICFR signatures predict HNSCC patients' ICB response but do not constantly predict patients' survival after ICB treatment.** The first ICFR signature is (B_{memory} - T_{reg}) / (T_{reg} + Plasma) (**A-D**), the second signature is B_{memory} / (T_{reg} + Plasma) (**E-H**). ROC curves of ICB response prediction using the first ICFR signature on the single-cell dataset (**A**) and the bulk dataset (**C**). Survival analysis using the first ICFR signature on the single-cell dataset (**B**) and the bulk dataset (**D**). ROC curves of ICB response prediction using the second ICFR signature on the single-cell dataset (**E**) and the bulk dataset (**G**). Survival analysis using the second ICFR signature on the single-cell dataset (**F**) and the bulk dataset (**H**). It should be noted that tumor ICFRs on the single-cell dataset are predicted from the blood, while the tumor ICFRs on the bulk dataset are calculated from deconvolution results using CIBERSORT.
