# Supplementary material for: Inferring Characteristics of the Tumor Immune Microenvironment of Patients with HNSCC from Single-Cell Transcriptomics of Peripheral Blood
Source: Cancer Res Commun. 2024 Sep 5;4(9):2335–48. doi: 10.1158/2767-9764.CRC-24-0092 (PMC11375407; doi:10.1158/2767-9764.CRC-24-0092)
Supplement: Supplementary Table 1 [file crc-24-0092_supplementary_table_1_suppst1.pdf]

Supplementary Table 1. Datasets used in this study.

Data source	Samples	Description	Purpose
GSE139324	26	Matched tumor-PBMC CD45+ scRNA-Seq	Model training
GSE200996	5	Matched tumor-PBMC CD45+ scRNA-Seq	Model validation
	27	PBMC CD45+ scRNA-Seq, patients’ ICB response	Signature identification
GSE159067	102	Bulk tumor RNA-Seq, patients’ overall survival and progression-free survival, ICB response	Signature validation
MSK-IMPACT	69	TMB, patients’ ICB response	Predictive power comparison
TCGA	520	Bulk tumor RNA-Seq, patients’ overall survival and progression-free survival	Survival analysis
