# Supplementary material for: Inferring Characteristics of the Tumor Immune Microenvironment of Patients with HNSCC from Single-Cell Transcriptomics of Peripheral Blood
Source: Cancer Res Commun. 2024 Sep 5;4(9):2335–48. doi: 10.1158/2767-9764.CRC-24-0092 (PMC11375407; doi:10.1158/2767-9764.CRC-24-0092)
Supplement: Supplementary Table 2 [file crc-24-0092_supplementary_table_2_suppst2.pdf]

Supplementary Table 2. Functional enrichment of predictable genes in the 11 immune cell types in the TME. The counts represent the number of immune cell types in which the corresponding significantly enriched term is present. The 15 most frequently present GO, KEGG, HALLMARK and REACTOME terms (all with FDR < 0.05) are shown, respectively.

GO term	Counts	KEGG term	Counts	HALLMARK term	Counts	REACTOME term	Counts
Adaptive immune response based on somatic recombination of immune receptors built from immunoglobulin superfamily domains	11	Cellular senescence	11	TNFA signaling via NFKB	11	Class_I MHC mediated antigen processing presentation	11
Aromatic compound catabolic process	11	Epstein-Barr virus infection	11	Allograft rejection	10	Neutrophil degranulation	11
Cellular nitrogen compound catabolic process	11	Graft-versus-host disease	11	MYC targets v1	10	Transcriptional regulation by TP53	11
Heterocycle catabolic process	11	Human T-cell leukemia virus 1 infection	11	Interferon gamma response	9	Activation of the mRNA upon binding of the cap binding complex and EIFS and subsequent binding to 43s	10
Leukocyte cell-cell adhesion	11	Allograft rejection	10	Interferon alpha response	8	Cellular response to starvation	10
Organic cyclic compound catabolic process	11	Antigen processing and presentation	10	Oxidative phosphorylation	8	Eukaryotic translation elongation	10
Positive regulation of T cell activation	11	Coronavirus disease - COVID-19	10	DNA repair	7	Eukaryotic translation initiation	10
Protein folding	11	Endocytosis	10	MTORC1 signaling	7	HIV infection	10
Response to endoplasmic reticulum stress	11	Kaposi sarcoma-associated herpesvirus infection	10	Unfolded protein response	7	Influenza infection	10
Response to virus	11	Phagosome	10	E2F targets	6	Interferon signaling	10
Ribosome assembly	11	Ribosome	10	G2M checkpoint	4	Metabolism of amino acids and derivatives	10
T cell activation	11	Salmonella infection	10	Mitotic spindle	4	mRNA splicing	10
Cytoplasmic translation	10	Spliceosome	10	Apoptosis	3	Nonsense mediated decay NMD	10
Establishment of protein localization to organelle	10	Th17 cell differentiation	10	Complement	3	Processing of capped intron containing pre mRNA	10
Histone modification	10	Type I diabetes mellitus	10	IL6 JAK STAT3 signaling	3	Regulation of expression of SLITS and ROBOS	10
